# N6-methyladenosine reader IMP2 stabilizes the ZFAS1/OLA1 axis and activates the Warburg effect: implication in colorectal cancer

**DOI:** 10.1186/s13045-021-01204-0

**Published:** 2021-11-07

**Authors:** Senxu Lu, Li Han, Xiaoyun Hu, Tong Sun, Dongping Xu, Yalun Li, Qiuchen Chen, Weifan Yao, Miao He, Zhenning Wang, Huizhe Wu, Minjie Wei

**Affiliations:** 1 Department of Pharmacology, School of Pharmacy, China Medical University, Shenyang, 110122 People’s Republic of China; 2 Liaoning Key Laboratory of Molecular Targeted Anti-Tumor Drug Development and Evaluation; Liaoning Cancer Immune Peptide Drug Engineering Technology Research Center; Key Laboratory of Precision Diagnosis and Treatment of Gastrointestinal Tumors, Ministry of Education; China Medical University, Shenyang, 110122 People’s Republic of China; 3 Shenyang Kangwei Medical Laboratory Analysis Co. LTD, Shenyang, Liaoning Province People’s Republic of China; 4 Department of Anorectal Surgery, First Affiliated Hospital of China Medical University, Shenyang, 110001 People’s Republic of China; 5 Department of Surgical Oncology and General Surgery, Key Laboratory of Precision Diagnosis and Treatment of Gastrointestinal Tumors, Ministry of Education, First Affiliated Hospital of China Medical University, Shenyang, 110001 People’s Republic of China

**Keywords:** m^6^A methylation, IMP2, ATP-hydrolyzing and glycolysis, ZFAS1, OLA1, Colorectal cancer

## Abstract

**Background:**

Accumulating evidence shows that N6-methyladenine (m^6^A) modulators contribute to the etiology and progression of colorectal cancer (CRC). However, the exact mechanisms of m^6^A reader involved in glycolytic metabolism remain vague. This article aimed to crosstalk the m^6^A reader with glycolytic metabolism and reveal a new mechanism for the progression of CRC.

**Methods:**

The relationship between candidate lncRNA and m^6^A reader was analyzed by bioinformatics, ISH and IHC assays. In vivo and in vitro studies (including MTT, CFA, trans-well, apoptosis, western blot, qRT-PCR and xenograft mouse models) were utilized to explore the biological functions of these indicators. Lactate detection, ATP activity detection and ECAR assays were used to verify the biological function of the downstream target. The bioinformatics, RNA stability, RIP experiments and RNA pull-down assays were used to explore the potential molecular mechanisms.

**Results:**

We identified that the crosstalk of the m^6^A reader IMP2 with long-noncoding RNA (lncRNA) ZFAS1 in an m^6^A modulation-dependent manner, subsequently augmented the recruitment of Obg-like ATPase 1 (OLA1) and adenosine triphosphate (ATP) hydrolysis and glycolysis during CRC proliferation and progression. Specifically, IMP2 and ZFAS1 are significantly overexpressed with elevated m^6^A levels in CRC cells and paired CRC cohorts (*n* = 144). These indicators could be independent biomarkers for CRC prognostic prediction. Notably, IMP2 regulated ZFAS1 expression and enhanced CRC cell proliferation, colony formation, and apoptosis inhibition; thus, it was oncogenic. Mechanistically, ZFAS1 is modified at adenosine +843 within the RGGAC/RRACH element in an m^6^A-dependent manner. Thus, direct interaction between the KH3–4 domain of IMP2 and ZFAS1 where IMP2 serves as a reader for m^6^A-modified ZFAS1 and promotes the RNA stability of ZFAS1 is critical for CRC development. More importantly, stabilized ZFAS1 recognizes the OBG-type functional domain of OLA1, which facilitated the exposure of ATP-binding sites (NVGKST, 32–37), enhanced its protein activity, and ultimately accelerated ATP hydrolysis and the Warburg effect.

**Conclusions:**

Our findings reveal a new cancer-promoting mechanism, that is, the critical modulation network underlying m^6^A readers stabilizes lncRNAs, and they jointly promote mitochondrial energy metabolism in the pathogenesis of CRC.

**Supplementary Information:**

The online version contains supplementary material available at 10.1186/s13045-021-01204-0.

## Background

N6-methyladenosine (m^6^A) was discovered in the early 1970s. It is the most abundant chemical modification of eukaryotic ribonucleic acid (RNA) including the messenger RNAs (mRNA) and long non-coding RNAs (lncRNAs) [1]. Reversible m^6^A modification of RNA plays critical roles in various aspects of the fate of RNA metabolism during tumor occurrence and progression, such as in pre-mRNA splicing, translocation, mRNA stability or decay, and lncRNA processing [2, 3]. A recent study recognized that dysregulation or alteration of cancer cell-intrinsic energy metabolism was one of the essential hallmarks of tumors, which was characterized in a glycolysis-dependent manner, and in turn, facilitated energy production by tumor cells [4]. However, the underlying mechanisms and regulatory network of m^6^A modification in tumor glycolysis during tumorigenesis have only a few reports, specifically for colorectal cancer (CRC). For example, Qing et al. revealed that R-2-hydroxyglutarate attenuated aerobic glycolysis in leukemia by targeting the FTO/m^6^A/PFKP/LDHB axis [5]; Liu et al. demonstrated that tumors could exploit FTO-mediated regulation of glycolytic metabolism to evade immune surveillance [6]; Wang et al. indicated that LncRNA LINRIS promoted aerobic glycolysis by stabilizing IGF2BP2 in CRC [7]. Thus, targeting interactions mediated by m^6^A epigenetic modification to metabolic glycolysis may contribute to providing better individual therapeutic interventions and may help in providing precise treatment for patients with CRC.

Accumulating evidence shows that dynamic m^6^A modification exerts its biological functions by predominantly recruiting specific m^6^A “readers”: class I readers such as the YT521-B homology (YTH) domain family (YTHDF1/2/3), class II readers such as hnRNP proteins (hnRNPC and hnRNPG), and the recently discovered class III readers such as the K homology (KH) domain family (IGF2BP1/2/3, also called IMP1/2/3) [8–10]. Among these, m^6^A readers IMP1/2/3 proteins are a well-established class of insulin growth factor-binding protein families; m^6^A modulator dysregulation has been recently associated with tumor aggressiveness and clinical prognostic survival in various cancers, including hepatocellular carcinoma (HCC), gastric cancer, pancreatic cancer, and CRC [11–14]. For example, Li et al. not only established a panel of potential biomarkers for prognostic prediction of patients with CRC but also uncovered a combined network of m^6^A modulators. “Reader” IMP2, “writer” METTL3, and “downstream target” SOX2 were highlighted in the novel m^6^A-dependent epigenetic regulation in CRC cells [15]. Another study reported that LINRIS blocked the K139 ubiquitination of IMP2 and prevented its degradation through the autophagy–lysosome pathway, consequently promoting CRC progression [7]. Although a recent study in pancreatic cancer deliberated that IGF2BP2 served as a reader for m^6^A-modified DANCR and stabilized DANCR RNA [16] comprehensive investigation is needed to explore the tumorigenesis and progression of CRC.

Recent studies have offered remarkable findings in the understanding of m^6^A readers such as the IMP family, which is delicately balanced to support cellular functions through various bioprocesses, including the translocation, translation, and degradation of RNAs. IMP1/2/3 proteins consist of similar RNA-binding domains, four C-terminal heterologous ribonucleoprotein KH domains, and two N-terminal RNA recognition motifs [17]. Functionally, the third and fourth KH (KH3–4) are identified as the core domains that are responsible for the direct recognition of m^6^A-containing RNAs (not limited to mRNAs) and enhancement of RNA stabilization by recruiting RNA co-stabilizers such as P-body, ELAV-like RNA-binding protein 1 (ELAVL1), and MATR3, thereby promoting the expression and translational efficiency of m^6^A-modified target [10]. Recent studies have shown that non-coding RNAs, particularly lncRNAs, exerted their functions through dynamic m^6^A modification in tumorigenesis [18]. Our previous study supports the overexpression of m^6^A writer METTL14 by LNC942 regulation, which depends on a direct binding pattern and is involved in breast cancer proliferation and progression [19]. Similarly, in pancreatic cancer cells, the m^6^A reader IMP2 shows remarkably enhanced expression and translation of lncRNA and DANCR by targeting its m^6^A-containing motif, which mutually accelerates the initiation and progression of pancreatic cancer [16]. However, the underlying mechanism of the m^6^A reader IMP2 that facilitates lncRNA stabilization in CRC tumorigenesis and progression remains unknown.

In this study, we found that the critical KH3–4 domain of m^6^A modulator IMP2 directly recognized specific m^6^A sites (“RGGAC/RRACH”) of ZFAS1 and promoted its stability and expression in an m^6^A-dependent manner. Subsequently, IMP2 stabilized ZFAS1 directly bound to the OBG-type functional domain of the OLA1 protein (OLA1, also called Obg-like ATPase 1, an adenosine triphosphate (ATP) hydrolase that binds to ATP through the amino acid site NVGKST [20]) to fully expose the ATP-binding site of OLA1 and ultimately enhanced the ATPase activity induced by OLA1. Notably, ZFAS1-activated OLA1 enhanced the ATP hydrolysis capacity and activated the Warburg effect. Thus, the proliferation and clone formation ability of CRC cells increased, and cell apoptosis was inhibited both in *vitro* and in *vivo*. Hence, we found a novel regulatory mechanism underlying the m^6^A reader IMP2, which was placed in the IMP2–ZFAS1–OLA1 signaling axis for CRC tumorigenesis and progression. Therefore, the findings of this study may help us understand the IMP2-stabilizing lncRNA–m^6^A epigenetic modification and associated glycolytic metabolism in CRC tumorigenesis and progression.

## Methods

### Tissue specimen collection

The human specimens included in this study were based on 144 pairs of clinical CRC tissues and matched adjacent-tumor controls from the First Hospital of Cancer Hospital of China Medical University and China Medical University between September 2014 and September 2015. All cases were diagnosed by histopathology. Patients with a history of other malignant tumors and who did not receive chemotherapy or radiation before surgery were excluded.

All patients provided written informed consent before enrollment in this study. Furthermore, the study was approved by the Medical Ethics Committee of China Medical University. These specimens were immediately snap-frozen in liquid nitrogen and stored at − 80 °C. The clinicopathological characteristics of these included patients are outlined in Additional file 1: Tables S5, S6, and S7.

### LncRNA–mRNA microarray assay

The *GeneChip*® Human Transcriptome Array 2.0 (HAT2.0, Affymetrix, Santa Clara, CA, USA) was explored, and it contained more than 6.0 million probes covering coding and non-coding transcripts of human genomes at Shanghai OE Biotech Technology Co, Ltd. (Shanghai, China). Raw data were deposited in GSE137511 (https://www.ncbi.nlm.nih.gov/geo/query/acc.cgi?acc=GSE137511). Databases including Ensembl, *UCSC*, *NONCODE*, *RefSeq*, *lncRNAdb*, *Vertebrate Genome Annotation*, *Mammalian Gene Collection*, and *Human Body Map lincRNAs* were selected to annotate the identified transcripts. Data were analyzed with the *Robust Multichip Analysis* algorithm using *Affymetrix* default analysis settings and global scaling as a normalization method.

### Gene expression analysis

GeneSpring (version 13.1; Agilent Technologies, Santa Clara, CA, USA) was employed to perform raw data analysis based on Biomarker technology (Beijing, China). Deferentially expressed genes were identified through fold change as well as *P* value calculated with *t*-test. The threshold of up- and down-regulated genes was set at fold change ≥ 2 and *P* ≤ 0.05. Finally, hierarchical clustering was performed to display the distinguishable expression pattern of genes from the eight samples included.

### Cell lines and Cell culture

All the cell lines (CRC cell lines including SW480, SW620, HCT116, SW48, RKO, CACO2, LOVO, HT29 and the normal human intestinal epithelial cell line HIEC) were purchased from Peking Union Medical College Cell Resource Center (PUMCCRC, Beijing, China). SW480, SW620, and SW48 cells were cultured in L15 medium (Hyclone, USA). HIEC, HCT116, LOVO, CACO2, RKO, and HEK293T cells were grown in Dulbecco’s Modified Eagle’s Medium (DMEM, Invitrogen, USA) and HT29 cells were maintained in 5A medium (5A, Invitrogen, USA) with 10% fetal bovine serum (PAA, Germany) at 37℃ with 5% CO2 atmosphere. In this study, all cells were determined to be free of mycoplasma contamination.

### Cell transfection

The short hairpin RNA (*shRNA*) for silencing ZFAS1 (*shZFAS1#1*, *shZFAS1#2*) and the negative control (*shNC*) were obtained from Genepharma (Shanghai, China). The *pcDNA-ZFAS1*, *pcDNA-IMP2*, *shIMP2#1*, *shIMP2#2* and blank vector (*NC*) were purchased by Genechem (Shanghai, China). All of the *shRNA* nucleotide sequences were listed in Additional file 1: Tables S10–S11. Plasmid extraction kit was purchased from Sangon Biotech (*Shanghai*, China). The cells were cultured on a small dish to a density of 60–70%, and then transfected with Lipofectamine 8000 (Invitrogen, USA) according to the manufacturer's instructions. Cells were collected 48 h after transfection and used for further experiments.

### RT-PCR assay and qPCR assay

Total RNA was extracted from CRC cells using TRIzol reagent according to the manufacturer’s instructions. Then cDNA was synthesized using the Reverse Transcription Kit (*TOYOBO*, Japan). qPCR assay was conducted using SYBR Green Real-time PCR Mix (Toyobo, Japan) and determined in triplicate. Data were normalized to GAPDH expression. The 2^*−ΔΔCt*^ method was calculated to represent the relative expression level of the gene. All primers were obtained from Sangon Biotech (Shanghai, China). The conditions of RNA reverse transcription and qPCR assay were listed in Additional file 1: Table S12. The primers used for RT-PCR and qPCR amplification were listed in Additional file 1: Table S13.

### Western blot analysis

The collected cell pellet was lysed with 1 × SDS buffer and centrifuged (13,000 rpm, 4 °C) for 10 min after sonication. After concentration detection, the extracted protein was separated by 8–12% SDS-PAGE and transferred to a polyvinylidene fluoride membrane (Millipore, Bedford, MA). Membranes were immunoblotted with anti-rabbit OLA1(HPA035790, 1:500, Sigma, USA), HK2 (2867S, 1:1000, CST, USA), PDHA1 (ab168379, 1:1000, *Abcam*, UK), m^6^A (ab151230, 1:1000, *Abcam*, UK), and anti-mouse GAPDH (1:2000, Zsbio, China) after sealed with 2% BSA, and the membranes were incubated with hybrid secondary antibody, the data was statistically collected by Fluor Chem V2.0 (Alpha Innotech Corp, USA).

### Tissue microarray (TMA) and immunohistochemistry (IHC)

TMA and IHC assays were performed as previously described [21]. Briefly, Paraffin donor blocks containing representative CRC tissues and tumor-adjacent controls were included by reviewing the hematoxylin and eosin-stained slides. A tissue core with a diameter of 1.5 mm was extracted from each donor block and accurately arranged into a new paraffin acceptor block with up to 55 cores using the Organization Microarrayer (Pathology Devices, USA). Sections (4 μm) were deparaffinized with xylene, rehydrated in a graded alcohol series, and washed in distilled water. Thereafter, sections were incubated in the primary antibody of IMP2 (11601-1-AP, 1:200, Proteintech, China), m^6^A (ab151230, 1:500, Abcam, UK), OLA1 (HPA035790, 1:50, Sigma, USA) overnight at 4 °C, followed by incubation with biotinylated secondary antibodies for 30 min at 37 °C. The slides were incubated with horseradish peroxidase coupled streptavidin for an additional 30 min (LSAB kit; Dako, Glostrup, Denmark), and stained with DAB (3, 3-diaminobenzidine). Sections were counterstained with hematoxylin, dehydrated, and mounted. Protein expression levels were observed and counted under a microscope (Eclipse 8i, Nikon, Japan) for subsequent analysis.

### LncRNA ISH assay

RNA ISH assay was performed strictly following the kit instructions (Boster, Wuhan, China). The sections were deparaffinized, deproteinized, and pre-hybridized with pre-hybridization solution at 42 °C for 2 h, then incubated with the DIG-labeled probe solution (Dilute 4 times with 1× PBS) at 37 ℃ overnight. The specific sequences of probes for ZFAS1 were showed in Additional file 1: Table S14. After stringent washing, the slides were exposed to a streptavidin-peroxidase reaction system and stained with DAB (Zsbio, Beijing, China) for 2 min. Then 0.1% Hematoxylin (Solarbio, Beijing, China) was used to counterstain the slides for 5 min. ZFAS1 expression levels were observed and counted under a microscope (Nikon, Tokyo, Japan).

### Cell proliferation assay

The cell proliferation viability was assessed by MTT assay (M8180, Solarbio, China). Cells were seeded on 96-well plates in triplicates at the density of 2000–4000 cells/100 μl. Thereafter, 20 μl of MTT solution was added and incubated at 37 °C for 3–4 h. The reaction liquid was abandoned and 150 μl of DMSO was added. Finally, the optical density 570 (OD_570_) absorbance values were obtained in triplicate and replicated 6 times.

### CFA

The transfected cells were seeded into 6-well plates (500 cells/well) for culturing for two weeks. After the medium was abandoned, visible colonies were washed with 1× PBS, fixed with 4% paraformaldehyde for 30 min, and stained with 0.1% crystal violet for 10 min. Finally, the number of colonies with more than 50 cells was calculated in this study.

### Flow cytometry assays

For apoptosis assays, Annexin V PE Apoptosis Kit (556547, BD Biosciences, Franklin Lakes, NJ, USA) was used. Cells were washed twice with cold phosphate-buffered saline, stained with PE-Annexin V and 7-AAD on ice, and subjected to flow cytometric analysis on a BD LSRFortessa analyzer (BD Biosciences).

### Trans-well assay

Cell migration assay was conducted with a 24-well insert Trans-well chambers (corning Costar, USA). The transfected cells (2 × 10^4^/well) were suspended in a serum-free medium and then added into the superior chamber. In contrast, the basal medium contained 10% fetal bovine serum. After 48 h, cells on the upper filter membrane were wiped off with cotton swabs. Chambers were washed with PBS, and then the cells on the lower filter membrane were fixed with 4% paraformaldehyde for 30 min and then washed with PBS. After staining with 0.1% crystal violet solution for 30 min, and rinsing with distilled water, intruded and migrated cells were randomly selected from 5 visual fields under the microscope to count the number of cells.

### IF assay

Briefly, cells were seeded into six-well plates containing glass coverslips, fixed with 4% paraformaldehyde, blocked with goat serum, and stained with anti-OLA1 antibodies (HPA035790, 1:50, Sigma, USA). Alexa Fluor anti-rabbit IgG (#4417, 1:500, Cell Signaling Technology, Danvers, MA, USA) were incubated at room temperature for 1 h. Cell nuclei were counterstained with DAPI (Beyotime Biotechnology, Shanghai, China). All image acquisition was processed using a Nikon C2 plus confocal microscope (Nikon, Japan).

### Co-localization of LncRNA and protein expression

Cells were cultured on cover slides and fixed normally following the steps of IF. Then RNA ISH assay was further performed following the kit instructions above except counterstaining with 0.1% Hematoxylin. Next, the cell was continued to stain with indicated OLA1 antibody, Alexa Fluor anti-rabbit IgG, and DAPI as immunofluorescence. Nikon C2 plus confocal microscope was used to obtain images (Nikon, Japan).

### ***m***^***6***^***A dot blot assay***

The total RNA was denatured in the three-fold volume of RNA incubation buffer (65.7% formamide, 7.77% formaldehyde, and 1.33 × MOPS) at 65 °C for 5 min, followed by chilling on ice and mixing with a one-fold volume of 20 × SSC. RNA samples were applied to Amersham Hybond-N^+^ membrane (GE Healthcare, Chicago, IL, USA) with a Bio-Dot Apparatus (BioRad, Hercules, CA, USA). After UV cross-linking, the membrane was stained with 0.02% methylene blue in 0.3 M sodium acetate. The membrane was then washed with 1× PBST buffer, blocked with 5% non-fat milk in PBST, and incubated with anti-m^6^A antibody (ab151230, 1:1000, Abcam, Cambridge, UK) overnight at 4 °C. After incubating with horseradish peroxidase-conjugated anti-rabbit IgG secondary antibody (Santa Cruz Biotechnology, CA, USA), the membrane was visualized using Fluor Chem V2.0 (Alpha Innotech Corp, CA, USA).

### RNA stability assay

HCT116 or SW620 cells were transfected with *sh*NC, *sh*IMP2#1, followed with treatment by actinomycin D (CAS#: A4262, Sigma) at a final concentration of 5 μg/mL for 2, 4, and 6 h. Total RNA was extracted and analyzed by qRT-PCR. Then, the calculation of RNA turnover rate and half-life (*t*_1/2_) of ZFAS1 and OLA1 were determined according to the previous publications. Since actinomycin D treatment results in transcription stalling, the change of RNA concentration at a given time (d*C*/d*t*) is proportional to the constant of RNA decay (*K*_decay_) and RNA concentration (*C*) as shown in the following equation:

$${\text{d}}C/{\text{d}}t = - K_{{{\text{decay}}}} C$$

Thus, the RNA degradation rate *K*_decay_ was estimated by:

$${\text{ln }}\left( {C/C_{0} } \right) = - K_{{{\text{decay}}}} t$$

When 50% of RNA is decayed, the equation below can be used to calculate RNA half-life (*t*_1/2_):

$${\text{ln}}\left( {{1}/{2}} \right) = - K_{{{\text{decay}}}} t_{{{1}/{2}}}$$

From where:

$$t_{{{1}/{2}}} = {\text{ln}}\,{2}/ - K_{{{\text{decay}}}}$$

### RNA pull-down assay

HEK293T cells with or without IMP2 knockdown were seeded in a 10-cm dish at 70%–80% confluency, cross-linked by UV and harvested by trypsinization. 40 μM of ZFAS1-wild biotin-labeled probe, ZFAS1-mutant biotin-labeled probe (specific sequences of probes for pull-down are shown Additional file 1: Table S15) were conjugated to Streptavidin agarose resin beads (Thermo Fisher Scientific, Waltham, MA, USA) by incubation for 4 h at 4 °C, respectively, washed three times, and incubated with pre-cleared protein extraction in RIP buffer at 4 °C overnight. After three washes with the RIP buffer, proteins were isolated with 40 μl 1 × SDS protein lysis at 95 °C for 10 min and centrifuged at 13,000g for 10 min. Input and co-immunoprecipitated proteins were analyzed by sodium dodecyl sulfate–polyacrylamide gel electrophoresis separation.

### RIP assay

HEK293T cells with or without IMP2 knockdown were seeded in 10-cm dish at 70%–80% confluency and harvested by trypsinization. The RIP kit (Geneseed Biotech, Guangzhou, China) was used to capture the antigen after the magnetic beads were connected to the m6A antibody or IgG antibody, RNAs were then extracted, and PCR was employed for verification.

### ATP content detection assay

After 48 h of transfection, the cells were collected, and ATP was extracted and detected with an ATP detection kit (SolarBio Life Science, Beijing, China) at a detection wavelength of 340 nm.

### LD content detection assay

The supernatant was collected 48 h after cell transfection, and after appropriate dilution, the absorbance was measured using an LD detection kit (BestBio, Wuhan, China) with a detection wavelength of 530 nm.

### ATPase activity detection assay

After 48 h of transfection, 5 × 10^6^ cells were taken and sonicated to extract the protein, and the ATPase activity was detected with an ATP detection kit (SolarBio Life Science, Beijing, China) at a detection wavelength of 660 nm.

### Extracellular acidification rate assay

HCT116 or SW620 cells (1.5 × 10^3^) were seeded in *XF*24 cell plates and then transfected. Cells were washed and placed in a 37 °C CO_2_-free incubator for 60 min. Thereafter, glucose, oligo, and 2-deoxyglucose were added, and the Seahorse Bioscience extracellular flux analyzer (Seahorse *XF*24 FluxPak, Agilent Technologies, North Billerica, MA, USA) was used for detection.

### Transmission electron microscopy

Collected cells were fixed in the electron microscope solution for 2 h and then sent to the School of Life Sciences of China Medical University for electron microscope analysis.

### Xenograft mouse experiment

The 4-week-old BALB/c-nu mice (half of the female and half of the male) were purchased from Shanghai Laboratory Animal Center (Shanghai, China). Before the experiments, the mice were acclimatized to the new environment for 1 week. They were randomized into groups with approximately equivalent numbers before tumor cell inoculation. The investigator was blinded to the group allocation of the animals during the experiment; 5 × 10^6^ HCT116 cells were injected subcutaneously into the right armpit region. All protocols followed the Regulations of Experimental Animal Administration issued by the Ministry of Science and Technology of the People’s Republic of China. If the tumors were visible, their weight and size were measured every 3 days. The mice’s weight and tumor growth were assessed every 5 days. In each group, the number of tumors or the tumor weight was evaluated in a blinded manner. The tumor volume was calculated by the equation *V* = 0.5 × *D* × *d* ^2^, where *V* is equal to the tumor volume, while *D* and *d* represent the longitudinal and latitudinal diameters, respectively. At 40 days after injection, the mice were sacrificed, and subcutaneous tumors were isolated and assessed. Moreover, the tumor tissues were fixed in 10% formalin for further experiment.

### Statistical analysis

In the present study, all of the statistical analysis was employed using SPSS 19.0 software package (SPSS Inc. Chicago, USA), GraphPad Prism 7 software (GraphPad, USA), and Microsoft office 2016 EXCEL (USA). *P* < 0.05 was considered statistically significant. *Pearson χ*^2^ or Fisher’s exact test was performed to find out the associations between the indicator expression and clinicopathological parameters in our included CRC patients. Linear regression analysis was explored to analyze the correlation of ZFAS1 with other indicators. Student’s *t*-test (two-tailed) and Wilcoxon or Welch’s *T-*test were used to comparing the significant differences of the paired and unpaired continuous variables between groups, presented as mean ± standard deviation (*s.d.*) or median (quartile).

For cell experiments study, the sample size was determined to be adequate based on the magnitude and consistency of measurable differences between groups, usually, the number is three or more. For the xenograft mice experiment, no statistical methods were used to predetermine sample size, which was determined based on previous experimental observations. The sample size for each experiment is indicated in the figure legend. No data were excluded from the analyses.

## Results

### Correlation of IMP1/2/3 expression with dysregulated lncRNAs in CRC cells and tissues

**Fig. 1:**
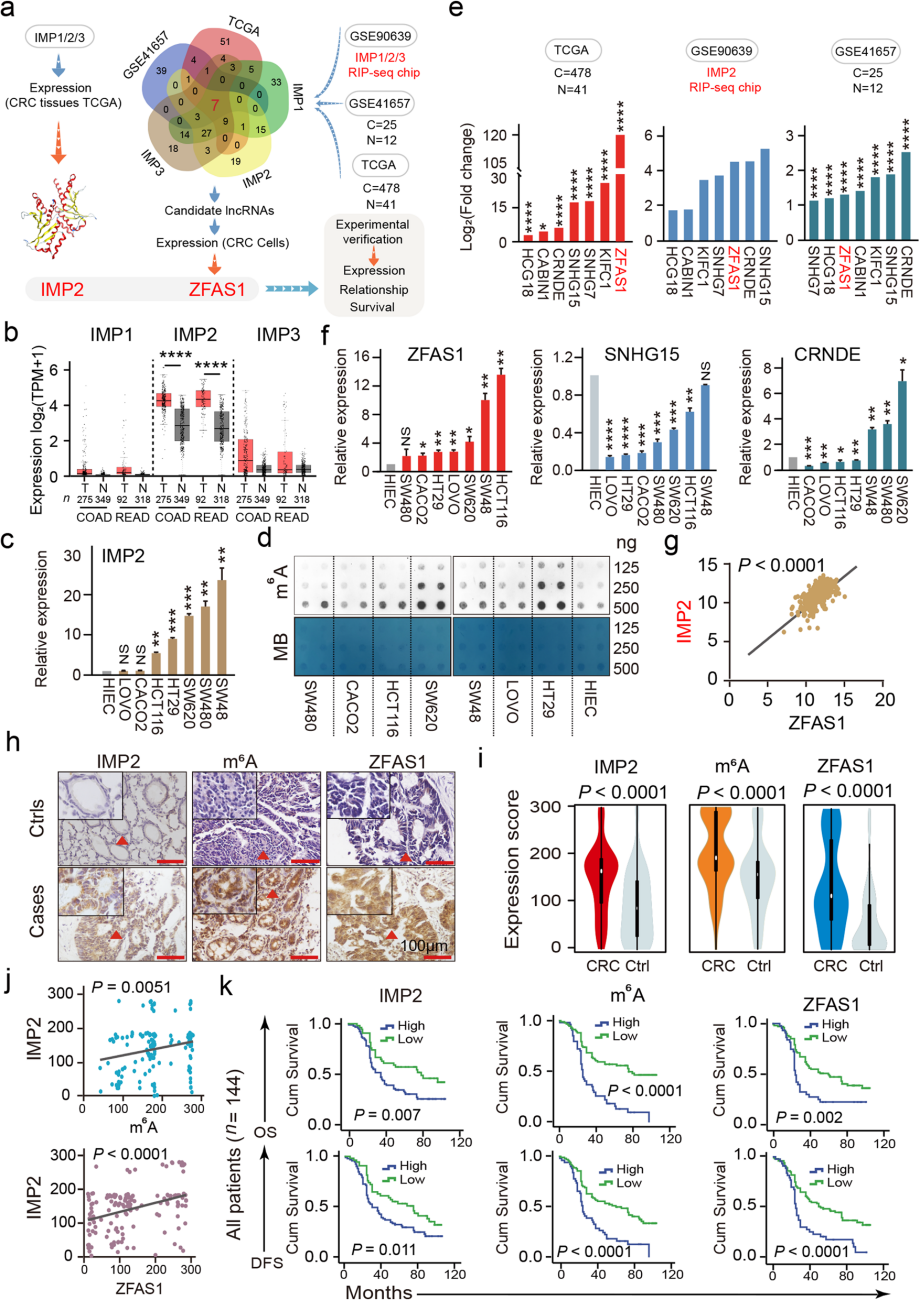
The relationship of IMP1/2/3 with lncRANs expression in CRC cells and patient tissues. **a** The screening strategy of IMP2 and its downstream target *ZFAS1*. **b** Expression of IMPs in COAD and READ tissues. **c** The RNA expression levels of *IMP2* in normal control cell HIEC and CRC cells including HCT116, SW620, SW480, LOVO, HT29, CACO2, and SW48 are detected by the qPCR assays.** d** The m^6^A expression levels in normal control cell HIEC and CRC cells including HCT116, SW620, SW480, LOVO, HT29, CACO2, and SW48 are detected by the m^6^A dot blot assay. **e** The expression of *ZFAS1*, *SNHG15*, *CRNDE*, *CABIN1*, *HCG18*, *KIFC1* and *SNHG7* in CRC in TCGA database and GEO database (GSE90639, GSE41657). **f** The RNA expression levels of *ZFAS1*, *CRNDE* and *SNHG15* in normal control cell HIEC and CRC cells including HCT116, SW620, SW480, LOVO, HT29, CACO2, and SW48 are detected by the qPCR assays. **g** Linear correlation pattern showing a positive relationship between the expression of *IMP2* and *ZFAS1* based on TCGA dataset. **h** ISH method detected the cellular localization and the expression of *ZFAS1*, and IHC assay determined m^6^A and IMP2 expression based on this included CRC patient tissues and matched tumor-adjacent controls (n = 144). The bar represents 100 μm. **i** Violin charts displaying the expression levels of IMP2, m^6^A, and *ZFAS1* in this included CRC cohort. Nonparametric tests and median (interquartile range) were shown. **j** Linear correlation pattern showing a positive relationship between the expression of IMP2, m^6^A, and *ZFAS1*. **k** Kaplan–Meier plot curves showing the association of IMP2 high/low expression, m^6^A high/low expression, and lncRNA *ZFAS1* high/low expression with the OS and DFS in this included CRC patients. Data were shown as mean ± s.d. of at least three independent experiments. *P < 0.05; **P < 0.01; ***P < 0.001; ****P < 0.0001 and ns no significance

M^6^A readers such as the IMP family affect the fates of mRNAs including RNA transcription, processing, translation, and metabolism in an m^6^A-dependent manner. However, how the IMP1/2/3 family recognizes lncRNAs has not been explored, particularly in CRC. Interestingly, in CRC tissues, only IMP2 was overexpressed, and its expression was much higher than those of IMP1 and IMP3 (http://gepia.cancer-pku.cn/, Fig. 1b). Similarly, IMP2 expression in seven CRC cell lines (LOVO, CACO2, SW48, SW480, HT29, HCT116, and SW620) is higher than that of human intestinal epithelial cells (HIEC) in colorectal epithelial cells (Fig. 1c). The results of the m^6^A dot blot assay demonstrated that the total m^6^A levels of CRC cell lines, especially in HCT116, SW620, and HT29 cell lines, were higher than that of HIEC in normal colorectal epithelial cells (Fig. 1d). This is also consistent with the function of IMP2, i.e., to stabilize RNA expression, which increases the m^6^A level of total RNA. Thereafter, we analyzed the m^6^A reader IMP1/2/3 RNA immunoprecipitation and sequencing (RIP-seq) chip GSE90639 (Additional file 1: Table S1), GSE41657 (Additional file 1: Table S2), and the Cancer Genome Atlas (TCGA) data (Additional file 1: Table S3). Finally, seven lncRNA candidates were obtained. Of which, ZFAS1, SNHG15, and CRNDE were significantly overexpressed (Fig. 1e). In CRC cells, ZFAS1 exhibited the most remarkable overexpression (Fig. 1f). Moreover, from the results of the Gene Expression Profiling Interactive Analysis website, differences in ZFAS1 expression in COAD and READ were more obvious than those of SNHG15 and CRNDE (Additional File 2: Fig. S1a). Consistently, our previous study [22] of lncRNA microarrays (GSE137511) also demonstrated significantly elevated levels of ZFAS1 (log_2_ FC = 6.65). Then, a correlation study was conducted between IMP1/2/3 and ZFAS1. The results presented a significantly positive correlation between ZFAS1 and IMP2 (*R* = 0.6476, *P* < 0.0001, Fig. 1g), and no linear correlation was observed between ZFAS1 and IMP1 or IMP3 (Additional File 2: Fig. S1b). Furthermore, the *ENCORI* platform (http://starbase.sysu.edu.cn/index.php) presented 111 supported binding sites between IMP2 and ZFAS1; however, only 28 and 35 binding sites were found between ZFAS1 and IMP1 or IMP3, respectively (Additional file 1: Table S4). These results lead us to explore the association of IMP2 with ZFAS1.

Thereafter, to investigate the potential roles of IMP2 in ZFAS1 expression and their associated m^6^A methylation levels, we, for the first time, performed tissue microarray (TMA), RNA in situ hybridization (ISH), and immunohistochemistry (IHC) assays to detect the expressions of IMP2, m^6^A, and ZFAS1 in our relatively large samples of paired CRC tissues and matched adjacent-tumor controls (*n* = 144) (Fig. 1h). For each index of low or high expression in our cohort, the cutoff values of the indicators were determined by receiver operating characteristic (ROC) curve assays (Additional file 2: Fig. S1c). Notably, expressions of IMP2, ZFAS1, and m^6^A methylation were dramatically increased in CRC tissues (Fig. 1i). Further correlation pattern analysis confirmed a positive linear relationship between IMP2 expression and ZFAS1 expression (*R* = 0.357, *P* < 0.0001) and m^6^A levels (*R* = 0.232, *P* = 0.0051) (Fig. 1j).

To further explore the clinical utility of the indicators as independent prognostic factors in this cohort, the log-rank test and multivariate Cox regression analyses were performed in our 144 pairs of CRC cases and adjacent-tumor control samples. Remarkably, the log-rank test for prognostic analysis revealed that an elevated IMP2 expression significantly shortened the overall survival (OS) (*P* = 0.007; high expression, MST = 36 months; low expression, MST = 80 months) and disease-free survival (DFS) (high expression, MST = 30 months; low expression, MST = 75 months; *P* = 0.011) (Fig. 1k, Additional file 2: Fig. S1d). Similarly, the higher expression of m^6^A methylation was significantly associated with poor OS (high expression, MST = 26 months; low expression, MST = 76 months; *P* = 0.0001) and DFS (high expression, MST = 26 months; low expression, MST = 62 months, *P* = 0.0001) (Fig. 1k, Additional file 2: Fig. S1dzz). Meanwhile, the OS of patients with CRC having a higher ZFAS1 expression was dramatically reduced (high expression, MST = 25 months; low expression, MST = 61 months, *P* = 0.002), similar to DFS, which was significantly reduced (high expression, MST = 25 months; low expression, MST = 52 months; *P* = 0.0001) (Fig. 1k, Additional file 2: Fig. S1d). Moreover, the interactive effects of IMP2, m^6^A, and ZFAS1 expressions on environmental factors and clinical variables were detected by unconditional logistic regression analysis adjusted for sex, age, tumor size, and tumor differentiation. The expressions of IMP2, m^6^A, and ZFAS1 significantly correlated with the survival status of patients with CRC including DFS and OS (Additional file 1: Tables S5, S6, and S7).

Altogether, these results indicated that IMP2 was overexpressed, accompanied by its correlated m^6^A methylation level and ZFAS1 expression in human CRC cells and tissues. These indicators also demonstrated potential functions as independent prognostic predictors for CRC evaluation and therapeutic biomarkers in the future.

### ***IMP2 promotes ZFAS1 stability by directly binding to the m***^***6***^***A site of ZFAS1 in CRC cells***

**Fig. 2:**
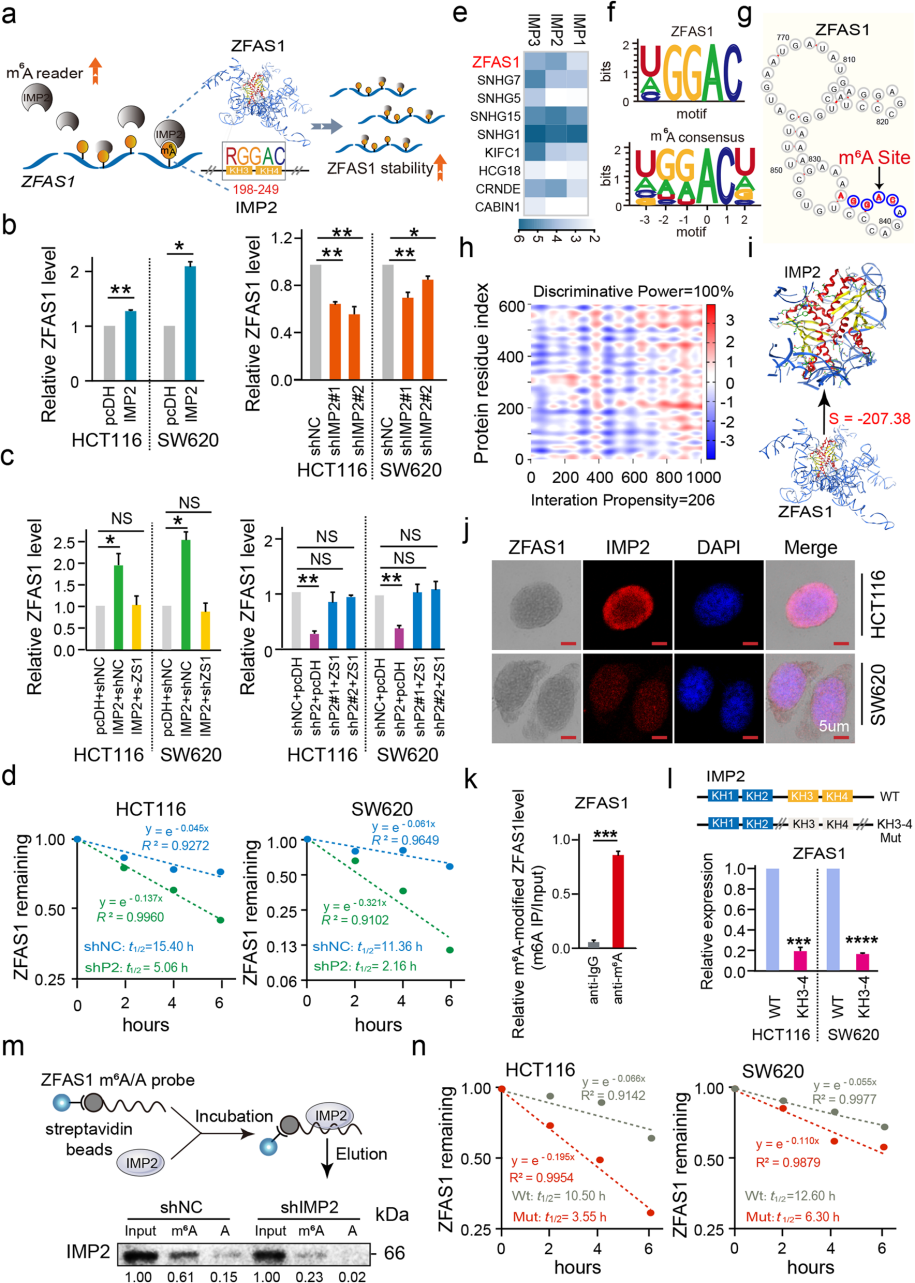
IMP2 promotes the stability of *ZFAS1* by directly binding to the m^6^A site of *ZFAS1*. **a** Schematic diagram of IMP2 directly binding to *ZFAS1* to improve *ZFAS1* stability. **b** The expression of *ZFAS1* after overexpression or silencing *IMP2* in HCT116 and SW620 cells by qPCR. **c** Rescue experiments detecting the *ZFAS1* expression levels after co-transfected with *IMP2* silencing and *ZFAS1* overexpression or *IMP2* overexpression and *ZFAS1* silencing vectors in HCT116 and SW620 cells assayed by the qPCR assay. **d** Reducing lncRNA half-life (t_1/2_) of *ZFAS1* decay after treated with *shIMP2* vectors in HCT116 and SW620 cells. **e** The heat map illustrating the differentially expressed lncRNAs in HEK293T. **f** CLIP database showing the motif of ZFAS1 targeting IMP2 and the motif of m^6^A site predicted by online software (http://www.starbase.sysu.edu.cn). **g** Cuilab (http://www.cuilab.cn/) online platform showing qualified m^6^A binding sites (RGGAC) of *ZFAS1*. **h** Bioinformatics online software predicting the specific binding sequence and sites of *ZFAS1* secondary structure and IMP2 protein (http://www.tartaglialab.com/). **i** MOE docking platform showing the specific docking sites between *ZFAS1* tertiary structure and IMP2 protein. **j** Co-localization of *ZFAS1* and IMP2 protein detected by the combination of ISH and IF assays in HCT116 and SW620 cells. Scale bar = 5 μm. **k** RIP followed by qPCR showed binding of the *ZFAS1* with anti-m^6^A in HEK293T cells. **l** The relative expression of *ZFAS1* after treated with *IMP2-WT* and *IMP2-Mut* (KH3-4) vectors in HCT116 and SW620 cells. **m** RNA pull-down followed by western blot showed in vitro binding of the *ZFAS1-Wild* and *ZFAS1-Mutant* probes with IMP2 protein after *IMP2* silencing in HEK293T cells. **n** Reducing lncRNA half-life (t_1/2_) of *ZFAS1* decay after treated with *ZFAS1-Mut* vectors in HCT116 and SW620 cells. Data were shown as mean ± s.d. of at least three independent experiments. *P < 0.05; **P < 0.01; *** *P* < 0.001; **** *P* < 0.0001 and ns no significance

In the above analysis of CRC cells, regardless of using bioinformatics data or experimental data, a correlation was found between IMP2 and ZFAS1. Thus, to explore the critical biological characteristics of IMP2-mediated m^6^A methylation stability and its associated ZFAS1 expression in CRC tumorigenesis and progression, we initially detected the expressions of IMP2 and ZFAS1 after interfering IMP2 expression in both HCT116 and SW620 CRC cells. Our results demonstrated that ectopic IMP2 expression significantly enhanced ZFAS1 and IMP2 expressions. Nevertheless, IMP2 knockdown inhibits ZFAS1 and IMP2 expressions in HCT116 and SW620 CRC cells assayed by quantitative polymerase chain reaction (qPCR) (Fig. 2b, Additional file 2: Fig. S2a). Moreover, the rescue experiment by upregulating ZFAS1 expression in IMP2 knockdown CRC cells demonstrated that ZFAS1 expression recovered, as detected by qPCR assay (Fig. 2c). More importantly, RNA stability assays further identified that the half-life of ZFAS1 RNA was reduced by IMP2 knockdown or by actinomycin D treatment at different time points (0, 2, 4, and 6 h) in HCT116 and SW620 CRC cells compared with the half-life in cells treated with an empty vector (negative control group) (HCT116: *sh*NC *t*_1/2_ = 15.40; *sh*IMP2 *t*_1/2_ = 5.06; SW620: *sh*NC *t*_1/2_ = 11.36; *sh*IMP2 *t*_1/2_ = 2.16, Fig. 2d).

These findings convinced us that IMP2 as an m^6^A reader has a stabilizing effect on ZFAS1. To verify the direct relationship between IMP2 and ZFAS1, we initially employed RIP-seq with FLAG-tagged IMP1/2/3 overexpression from the GSE90639 dataset (https://www.ncbi.nlm.nih.gov/geo/query/acc.cgi?acc=GSE90639) and demonstrated that ectopic IMP2 expression dramatically enriched endogenous ZFAS1 compared with the input control (log_2_FC = 4.59) in HEK293T cells (Fig. 2e, Additional file 1: Table S1). Moreover, RNA-binding protein datasets supported that the IMP2 protein harbored a specific binding domain that recognizes the m^6^A RGGAC/RRACH motif within the *ZFAS1* sequence obtained from the *ENCORI* platform [23] (http://www.starbase.sysu.edu.cn) (Fig. 2f). Consistently, we successfully found four qualified m^6^A-binding RGGAC motifs and one m^6^A-binding RAGAC motif within the *ZFAS1* gene through the Cuilab (http://www.cuilab.cn/) online platform (Fig. 2g and Additional file 2: Fig. S2b). To further elucidate the critical domain of the IMP2 protein involved in stabilizing ZFAS1 expression and CRC cell fate, the *RNAfold* (http://rna.tbi.univie.ac.at/cgi-bin/RNAWebSuite/RNAfold.cgi) webserver was explored to predict the secondary structure of ZFAS1 (Additional file 2: Fig. S2c). The *catRAPID* (http://s.tartaglialab.com/page/catrapid_group) and Molecular Operating Environment (MOE) platforms were also utilized to determine the direct binding domains at both secondary and tertiary structural levels (Fig. 2h, i). The sequence “RGGAC/RRACH” (+833 to +869) of ZFAS1 exhibited remarkable interaction propensity (*value* = 206) and discriminative power (100%) with the IMP2 KH3–4 domain based on the protein sequence (+198 to  +249), and their interaction strength is 98% (Fig. 2h and Additional file 2: Fig. S2d, e). The annotated or putative RNA-binding domains of IMP2 protein are shown in Fig. S2f. Notably, the MOE software analyzed the recognizing motif (RGGAC/RRACH) of ZFAS1 interacting with the IMP2 KH3–4 amino acid domain (*SVM classifier* =  − 207.38) (Fig. 2i). To verify the reliability of the binding sites of IMP2 and ZFAS1, ISH and immunofluorescence (IF) assay for colocalization were performed and the consistent distributions of ZFAS1 and IMP2 expressions were confirmed, with the main locus in the cytoplasm and some parts in the nucleus of HCT116 and SW620 CRC cells (Fig. 2j). Furthermore, the RIP assay identified that enriched ZFAS1 expression (the enrichment ratio is 51.35%) was obtained from dragging after incubation with the m^6^A antibody compared with the input group (Fig. 2k). Moreover, we synthesized the biotin-labeled ZFAS1 probe containing the RGGAC/RRACH recognizing motif with the IMP2 protein (ZFAS1 m^6^A-WT) or containing a corresponding mutant sequence (ZFAS1 m^6^A-Mut) (Fig. 2m). Thereafter, RNA pull-down assays demonstrated that the ZFAS1 m^6^A-WT probe, but not ZFAS1 m^6^A-Mut, significantly pulled down the IMP2 protein, and this enrichment was further significantly reduced after IMP2 knockdown (Fig. 2m), indicating a direct binding interaction by harboring the m^6^A recognizing motif with the IMP2 protein-specific domain. To confirm the direct interaction of the IMP2 KH3–4 domain on ZFAS1 achieved by recognizing the m^6^A motif, qPCR assay in HEK-293 T cells was performed after treatment with IMP2 KH3–4 WT or Mut vectors. A significantly reduced expression of ZFAS1 was observed after treatment with IMP2 KH3–4 Mut vectors when compared with the IMP2 WT group (Fig. 2l). Thereafter, we mutated the m^6^A site (+833 to +869) on ZFAS1 and tested the stability of ZFAS1, which was also significantly reduced (HCT116: WT *t*_1/2_ = 10.50; Mut *t*_1/2_ = 3.55; SW620: WT *t*_1/2_ = 12.60; Mut *t*_1/2_ = 6.30, Fig. 2n). So far, we have confirmed that IMP2 directly binds to the m^6^A site on ZFAS1 through KH3–4 and enhances the stability of ZFAS1 (Fig. 2a).

### IMP2 mediates biological characteristics by regulating ZFAS1 expression in CRC cells

**Fig. 3:**
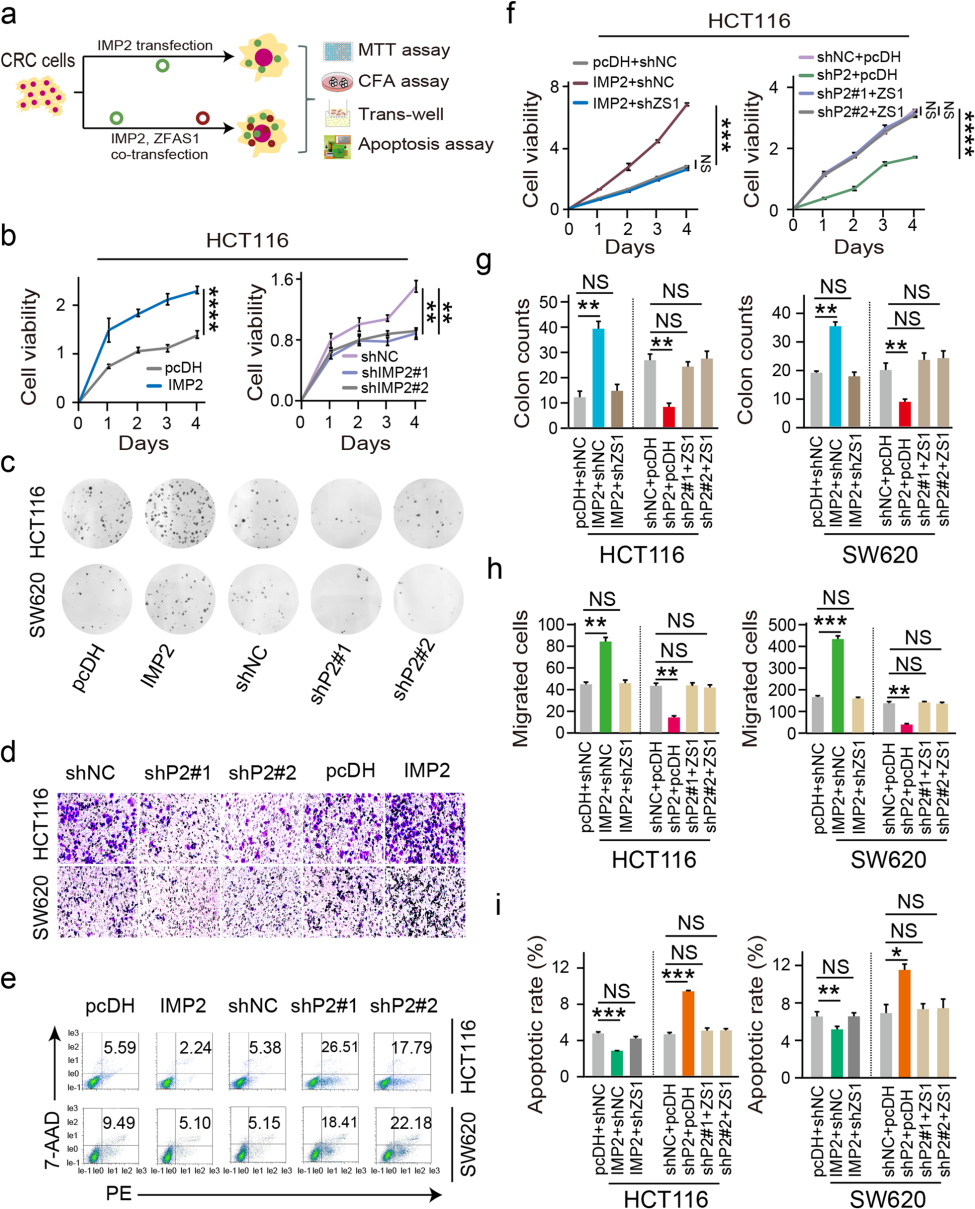
IMP2 mediates biological characteristics by regulating *ZFAS1* expression in CRC cells. **a** Flow-chart for detecting CRC phenotypes after single intervention with *IMP2* or simultaneous intervention with *IMP2* and *ZFAS1*. **b** MTT assay was performed to identify the cell viability upon *IMP2* silencing or overexpressing in HCT116 cells. **c** CFA displaying the colony-forming abilities in both HCT116 and SW620 cells after ectopic or knockdown *IMP2*. **d** The migration ability was determined after ectopic or knockdown *IMP2* in HCT116 and SW620 cells. **e** The percentage (%) of cell apoptosis was detected upon *IMP2* overexpressing or silencing in HCT116 and SW620 cells by Flow cytometry. **f** Rescue experiments detecting the cell viability treated by co-transfected *IMP2* silencing and *ZFAS1* overexpression/*IMP2* overexpression and *ZFAS1* silencing vectors in HCT116 cells assayed by MTT assay. **g, h, i** Rescue experiments detecting the colony-forming abilities, migration capacity, and cellular apoptotic rates after co-transfected with *IMP2* silencing and *ZFAS1* overexpression or *IMP2* overexpression and *ZFAS1* silencing vectors in both HCT116 and SW620 cells assayed by CFA method, trans-well assays, flow cytometry, respectively. Data were shown as mean ± s.d. of at least three independent experiments. Data were shown as mean ± s.d. of at least three independent experiments. *P < 0.05; **P < 0.01; *** *P* < 0.001; **** *P* < 0.0001 and ns no significance

In this study, to further clarify the regulation pattern of IMP2 and ZFAS1 in CRC cell characteristics, we first analyzed the cell proliferation ability and colony formation capacity after interfering IMP2 expression, as detected by the MTT assay (Fig. 3b) and colony formation assays (CFA) (Fig. 3c) in HCT116 and SW620 CRC cells, respectively. As expected, ectopic IMP2 expression significantly promoted the cell growth and colony formation ability of HCT116 and SW620 CRC cells, whereas IMP2 depletion suppressed the cell proliferative and colonic abilities of these two types of CRC cells (*P* < 0.05, Fig. 3b, c and Additional file 2: Fig. S3a, b). Furthermore, the trans-well assay was conducted to detect the invasive abilities, which revealed that migrated cells were significantly suppressed while silencing IMP2. Instead, IMP2 overexpression dramatically elevated the invasive ability of HCT116 and SW620 CRC cells (*P* < 0.05, Fig. 3d). Moreover, IMP2 knockdown increased the cellular apoptosis rate. Nevertheless, IMP2 overexpression substantially inhibited the apoptosis rate in both HCT116 and SW620 CRC cells determined by flow cytometry-PE/7AAD double staining (Fig. 3e, and Additional file 2: Fig. S3c).

To further establish the influence of IMP2 by regulating ZFAS1 expression and their possible biological function in CRC cells, rescue experiments were performed by enhancing ZFAS1 expression in IMP2 knockdown CRC cells. Primarily, the rescue experiments revealed that ZFAS1 overexpression reversed the facilitated effect on CRC molecular characteristics including cell proliferation ability, cell colony formation capacity, cell migration ability, and apoptotic rates in both HCT116 and SW620 CRC cells after MTT assay (Fig. 3f, and Additional file 2: Fig. S3d), CFA assay (Fig. 3g and Additional file 2: Fig. S3e), trans-well assay (Fig. 3h and Additional file 2: Fig. S3f), and flow cytometry analysis (Fig. 3i and Additional file 2: Fig. S3g), respectively. Thus, these results supported that IMP2 promotes CRC cell proliferation, inhibits CRC cell apoptosis, and promotes cell invasion and metastasis by promoting ZFAS1stability and expression, while the effect of IMP2 on cell phenotype can be restored by regulating ZFAS1 expression.

### Identification of OLA1 as the critical target of ZFAS1 stabilized by IMP2 in CRC cells

**Fig. 4:**
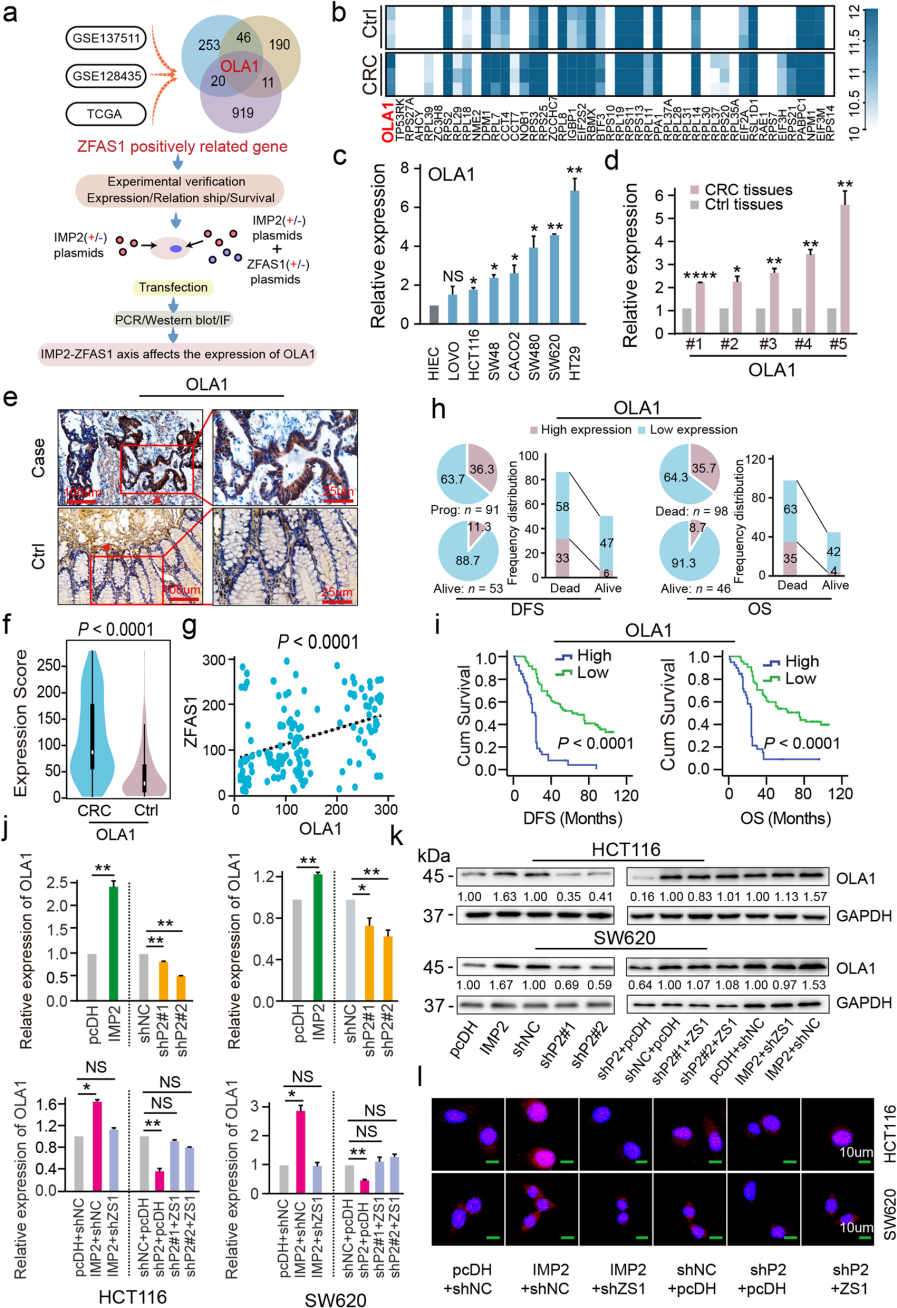
OLA1 is the critical downstream target of *ZFAS1* stabilized by IMP2 in CRC cells. **a** Flow chart of *ZFAS1* downstream target OLA1 screening and validation of the correlation between *ZFAS1* and OLA1 **b** The heat map illustrating the differentially expressed mRNAs in matched tumor-adjacent controls and matched CRC samples (*P* < 0.05). **c** The RNA expression levels of *OLA1* in normal control HIEC cells and CRC cells including HCT116, SW620, SW480, LOVO, HT29, CACO2, and SW48 are detected by qPCR assays. **d** The RNA expression levels of *OLA1* in paired tumor-adjacent control tissues and CRC samples (*n* = 5) detected by qPCR assays. **e** IHC assays displaying OLA1 expression in our included cohorts (*n* = 144). The bar represents 100 μm. **f** Violin charts displaying the expression levels of OLA1 in this included CRC cohort. Nonparametric tests and median (interquartile range) were shown. **g** Linear correlation pattern showing a positive relationship between the expression of *ZFAS1* and OLA1. **h** Pie chart and bar chart showing the frequency and percentage of OLA1 high/low expression patients in DFS/OS status is dead/alive patients. **i** Kaplan–Meier plot curves showing the association of OLA1 high/low expression with the OS and DFS in this included CRC patients. **j** The expression of *OLA1* after interfering with *IMP2* alone or interfering with *IMP2* and *ZFAS1* at the same time in CRC cells by qPCR. **k** Western blot detecting the protein levels of OLA1 after interfering with *IMP2* alone or interfering with *IMP2* and *ZFAS1* at the same time in CRC cells. **l** IF method illustrating the protein levels of OLA1 after interfering with *IMP2* alone or interfering with *IMP2* and *ZFAS1* at the same time in CRC cells. Data were shown as mean ± s.d. of at least three independent experiments. *P < 0.05; **P < 0.01; *** *P* < 0.001; **** *P* < 0.0001 and ns no significance

To excavate the downstream targets of ZFAS1 that affect the CRC cellular phenotype and function, a series of related functional investigations were performed in CRC cells. First, the potential downstream targets positively associated with ZFAS1 overexpression in GSE137511 were enriched by heatmap cluster analysis (Fig. 4b, Additional file 1: Table S8) and CRC samples were further expanded by combining GSE128435 and TCGA datasets (Fig. 4a). The mitochondrial energy metabolism-associated gene *OLA1* was exactly present in the intersection of these co-expression targets (Fig. 4a). Further examination results identified that OLA1 was dramatically overexpressed in seven CRC cell lines, including SW480, HT29, LOVO, CACO2, SW48, HCT116, and SW620, compared with HIEC control cells detected by qPCR assays (Fig. 4c). Similarly, an elevated OLA1 level was further confirmed in paired CRC tissues and compared with that of the matched tumor-adjacent controls (*n* = 5) using qPCR assays (Fig. 4d). OLA1 is also highly expressed in various tumor types including CRC (Additional file 2: Fig. S4a), and the expressions of OLA1 and ZFAS1 in CRC tissues are highly consistent (Additional file 2: Fig. S4b).

Thereafter, TMA and IHC assays were employed to detect OLA1 expression levels (Fig. 4e), and the ROC curve method was implemented to assess the low/high expression of OLA1 based on the cutoff value used in our CRC cohort (*n* = 144) (Additional file 2: Fig. S4c). As expected, marked OLA1 levels were observed in CRC tissues compared with those in matched tumor-adjacent controls, which exhibited the properties of an oncogenic gene (Fig. 4e, f). Furthermore, a positive linear correlation was confirmed between OLA1 expression and ZFAS1 expression (*R* = 0.361, *P* < 0.0001) (Fig. 4g). Similarly, in TCGA, OLA1 and ZFAS1 expressions in CRC tissues are highly consistent, showing positive linear correlation (*R*^2^ = 0.27, *P* < 0.0001) (Additional file 2: Fig. S4d). Notably, the correlation of the high/low expressions of OLA1 with the clinical outcomes or clinicopathological variables of patients with CRC was further explored, and the independent prognostic value of OLA1 was confirmed in this cohort. Specifically, the correlation analysis with clinicopathological parameters confirmed that the proportion of patients with low OLA1 expression was higher among surviving patients with DFS and OS status (Fig. 4h, Additional file 1: Table S9). Strikingly, high OLA1 expression significantly shortened the DFS time (high expression, MST = 23 months; low expression, MST = 63 months, *P* < 0.0001) and OS time (high expression, MST = 24 months; low expression, MST = 75 months, *P* < 0.0001) of patients with CRC (Fig. 4i). To verify that OLA1 is indeed the downstream target of ZFAS1 which is stabilized by IMP2, the mRNA and protein expressions of OLA1 were tested after interference with IMP2. Interestingly, IMP2 silencing/overexpression not only decreased/increased the expression of OLA1 mRNA, but also decreased/increased the expression of OLA1. However, the effect of IMP2 on OLA1 can be completely reversed by ZFAS1 (Fig. 4j–l, Additional file 2: Fig. S5e). Therefore, these findings suggested that OLA1 was positively regulated by m^6^A-modified and m^6^A-stabilized ZFAS1 expression in human CRC cells and tissues. The further prognostic evaluation indicated that OLA1 can be a potential biomarker for the prediction of CRC clinical outcomes.

### ZFAS1 enhances OLA1 activity and activates glycolysis by binding to the OBG-type functional domain of OLA1

**Fig. 5:**
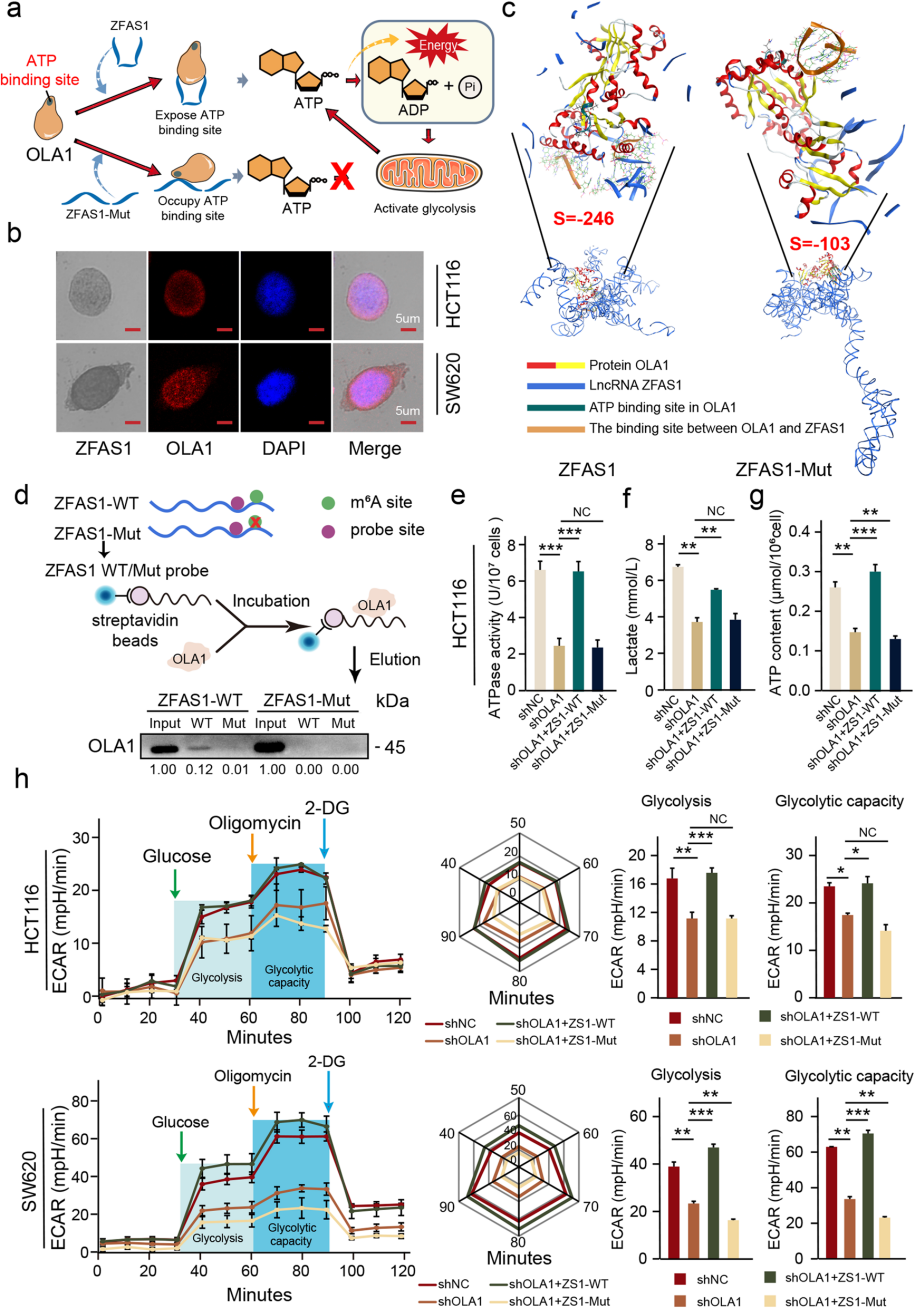
*ZFAS1* enhances OLA1 activity and activates glycolysis by binding to the OBG-type domain of OLA1. **a** Schematic diagram of *ZFAS1* directly binding to OLA1 to enhance its ATPase activity and ultimately promote ATP hydrolysis and glycolysis. **b** Co-localization of *ZFAS1* and OLA1 protein detected by the combination of ISH and IF assays in HCT116 and SW620 cells. Scale bar = 5 μm. **c** MOE verified that *ZFAS1-Wild* directly binds OLA1 protein and exposes the ATP-binding site of OLA1 protein. **d** RNA pull-down followed by western blot showed in vitro binding of the *ZFAS1-Wild* and *ZFAS1-Mutant* probes with OLA1 protein after *ZFAS1* mutation in HEK293T cells. **e** Rescue experiments determining the ATPase activity of OLA1 after co-transfected with *shOLA1* and *ZFAS1-Wild/ZFAS1-Mutant* vectors in HCT116 cells. **f**, **g** The content of lactate or ATP in the cell supernatant after co-transfected with *shOLA1* and *ZFAS1-Wild/ZFAS1-Mutant* vectors in HCT116 cells. **h** ECAR experiment verifying the effects of OLA1 and *ZFAS1-Wild/ZFSA1-Mutant* on glycolysis of CRC cells. Data were shown as mean ± s.d. of at least three independent experiments. *P < 0.05; **P < 0.01; *** *P* < 0.001; **** *P* < 0.0001 and ns no significance

In previous studies, we have determined that there is a positive correlation between ZFAS1 and OLA1. However, we have not yet clarified the interaction pathway between ZFAS1 and OLA1. To this end, we initially demonstrated the colocalization of these indicators in CRC cells through ISH and IF assay. Cellular colocalization indicated that OLA1 and ZFAS1 are consistently distributed not only in the cytoplasm but also in the nucleus of HCT116 and SW620 CRC cells (Fig. 5b). To determine whether ZFAS1 has a direct binding site with OLA1 and to examine the effect of the critical m^6^A site in ZFAS1 on OLA1, we substituted three A bases (+833 to +869) in ZFAS1 with U bases and re-predicted the tertiary structure of ZFAS1. Then, we docked ZFAS1-WT and ZFAS1-Mut with OLA1 by MOE. Interestingly, both ZFAS1-WT and ZFAS1-Mut are combined with the OBG-type functional domain of OLA1. However, the binding strength of ZFAS1-WT and OLA1 (*S* =  − 246) is much higher than that of ZFAS1-Mut and OLA1 (*S* =  − 103). Moreover, the combination of ZFAS1-WT and OLA1 fully exposes the ATP-binding site of OLA1 (NVGKST, 32–37), while the combination of ZFAS1-Mut and OLA1 invades the binding sites of OLA1 and ATP (Fig. 5c). To explore the internal mechanism of ZFAS1 and OLA1, we synthesized the biotin-labeled ZFAS1 probe containing the ZFAS1-OLA1 binding motif (ZFAS1-WT) or containing a corresponding mutant sequence (ZFAS1-Mut). Thereafter, the RNA pull-down assays demonstrated that the ZFAS1-WT probe, but not ZFAS1-Mut, significantly pulled down OLA1, and when the m^6^A site on ZFAS1 (+833 to +869) was mutated, the ZFAS1-WT probe could no longer pull down OLA1 (Fig. 5d). All results indicate a direct binding interaction (+732 to +754) by harboring the ZFAS1–OLA1 binding motif with the OLA1 protein-specific domain. However, the change in the three-dimensional structure of ZFAS1 caused by the mutation of the key m^6^A site on ZFAS1 can directly disrupt the binding of ZFAS1 and OLA1. Moreover, a reduction in OLA1 expression resulted in the decrease of its ATPase activity, and ZFAS1 overexpression could restore the ATPase activity of OLA1. However, when the key m^6^A site on ZFAS1 was mutated, the restoration effect of ZFAS1 on OLA1 ATPase activity disappeared (Fig. 5e, Additional file 2: Fig. S5a). This also proves that the promotion effect of ZFAS1 on OLA1 ATPase activity hinges on the combination of ZFAS1 and OLA1 to expose the ATP-binding site of OLA1.

To explore the effect of changes in OLA1 ATPase activity on the energy metabolism of CRC cells, we examined the contents of lactate and ATP in HCT116 and SW620 CRC cells. Interestingly, the decrease in OLA1 expression could not only reduce lactate accumulation in CRC cells but also reduce ATP content in cells; however, ZFAS1-WT, but not ZFAS1-Mut, could restore the effects of OLA1 deficiency (Fig. 5f, g, Additional file 2: Fig. S5b, c). The results contradict the ATP hydrolysis function of OLA1, which inevitably makes us wonder whether the main ATP production pathway is blocked. To verify this conjecture, an extracellular acidification rate assay was performed to detect the effect of changes in OLA1 on the glycolytic ability of CRC cells. The results are not surprising. The reduction of OLA1 expression could significantly reduce the glycolytic ability of CRC cells, while ZFAS1-WT, but not ZFAS1-Mut, could restore the effect of reduced OLA1 expression on the reduction of the glycolytic ability of CRC cells (Fig. 5h).

In conclusion, ZFAS1 can directly bind to the OBG-type functional domain of OLA1 to expose the ATP-binding site on OLA1, thereby enhancing the ATP hydrolysis ability of OLA1. The enhanced ATP hydrolysis ability of OLA1 promoted the accumulation of lactic acid and the release of the raw materials of ATP synthesis, thereby activating the glycolytic pathway of CRC cells, and ultimately promoted the release of energy in CRC (Fig. 5a).

### Mitochondrial energy metabolism was affected by the IMP2–ZFAS1–OLA1 signaling axis

**Fig. 6:**
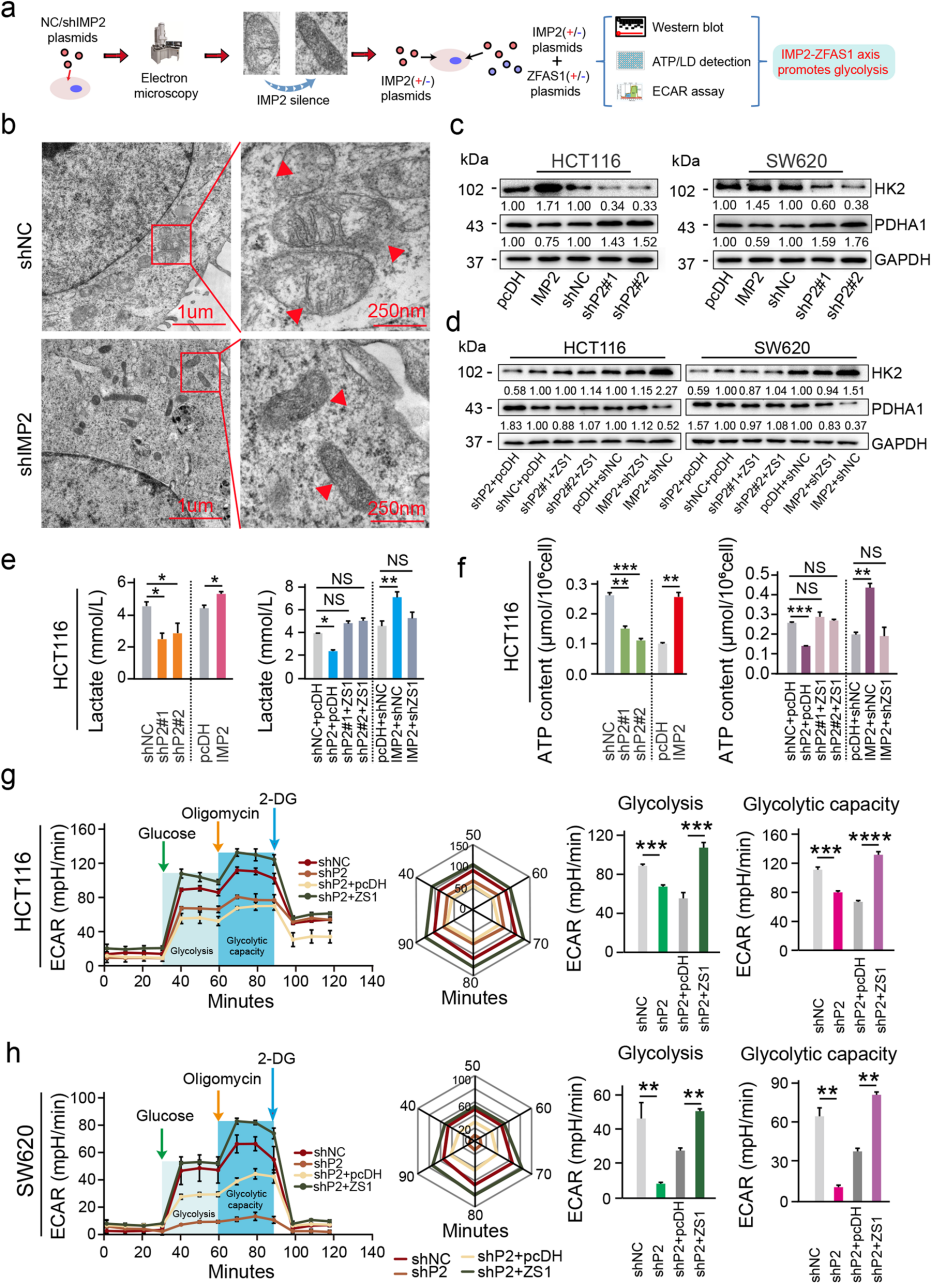
Mitochondrial energy metabolism was affected by the IMP2-*ZFAS1*-OLA1 signaling axis. **a** Flow chart of validation of IMP2-*ZFAS1*-OLA1 axis on energy metabolism. **b** Morphology of mitochondria under electron microscope after knockdown *IMP2* in HCT116 cells. **c** Western blot detecting the protein levels of mitochondrial associated protein HK2 and PDHA1 expression after interfering with *IMP2* expression in both HCT116 and SW620 cells. **d** Western blot assays detecting the protein levels of mitochondrial associated protein HK2 and PDHA1 after co-transfected with *shIMP2* upon *ZFAS1* overexpression or *IMP2* overexpression and *shZFAS1* vectors in HCT116 and SW620 cells. **e, f** Rescue experiments detecting the lactate content and ATP content treated by co-transfected with *shIMP2* upon *ZFAS1* overexpression or *IMP2* overexpression and *shZFAS1* vectors in HCT116 cells. **g** ECAR experiment verifying the effects of IMP2 and *ZFAS1* on glycolysis of CRC cells. Data were shown as mean ± s.d. of at least three independent experiments. *P < 0.05; **P < 0.01; *** *P* < 0.001; **** *P* < 0.0001 and ns no significance

**Fig. 7:**
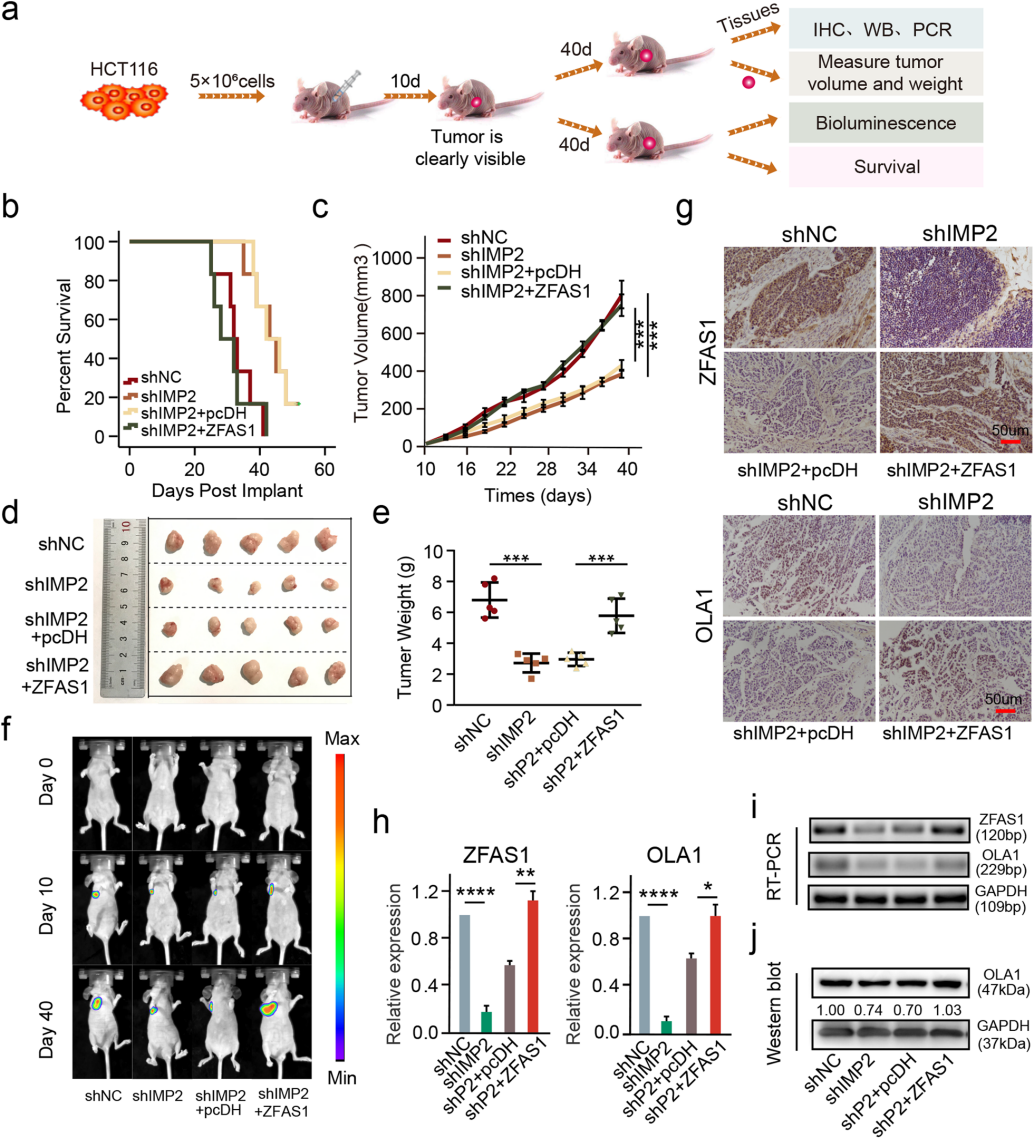
IMP2 promotes cell proliferation by stabilizing *ZFAS1 *in vivo. **a** Schematic diagram of xenografts in BALB/c nude mice by inoculating HCT116 cells at their right armpits that were stably co-transfected with *shNC*, *shIMP2*, *shIMP2* + *pcDH*, *shIMP2* + *ZFAS1*, respectively. **b** The survival time for each group xenografts in nude mice. **c** Mean volumes of xenografts on different days in nude mice. Data are shown as mean ± s.d., n = 5 for each group. **d** Representative xenografts are shown when excised on the 40th day. **e** Mean weight of xenografts for each group was determined on the 40th day. Data are shown as mean ± s.d., *n* = 5 for each group. **f** Tumor size of xenograft tumor at day 0, day 10, and day 40. **g** ISH and IHC assays were performed to determine the protein expression of *ZFAS1* and OLA1 above each group. **h, i** qPCR and RT-PCR assays were performed to determine the RNA expression of *ZFAS1* and *OLA1* in each group. **j** Western blot assay detecting the protein expression of OLA1 upon each group. The groups were as follows: *shNC* (empty vector); *shIMP2*; *shIMP2* + *pcDH* (co-transfected with *shIMP2* and *pcDH* empty vector); *shIMP2* + *ZFAS1* (co-transfected with *shIMP2* and *pcDH-ZFAS1*). Data were shown as mean ± s.d. of at least three independent experiments. *P < 0.05; **P < 0.01; *** *P* < 0.001; **** *P* < 0.0001 and ns no significance

To further elucidate the critical functions in the IMP2-stabilized signaling axis involved in mitochondrial energy metabolism during CRC progression, we first observed under an electron microscope the morphological changes of the mitochondria after IMP2 was silenced. As a result, in CRC cells, the mitochondria were markedly swollen and damaged and the morphology of the mitochondria returned to normal after IMP2 was silenced (Fig. 6b). That is, in CRC, most cells can only depend on glycolysis for energy, and after IMP2 silencing, the ratio of glycolysis energy for CRC cells will be significantly reduced. To verify our conjecture, we initially intervened with IMP2 and detected the expression of HK2 (key protein for glycolysis) and PDHA1 (key protein for oxidative phosphorylation). Consistently, IMP2 overexpression promoted the HK2 expression and inhibited PDHA1 expression (Fig. 6c). Nevertheless, IMP2 silencing can significantly decrease HK2 expression and increase PDHA1 expression in HCT116 and SW620 CRC cells (Fig. 6c). Moreover, the expression levels of HK2 and PDHA1 were restored after ZFAS1 silencing/overexpression with IMP2 overexpression/silencing in CRC cells (Fig. 7d). Thereafter, alterations in the hydrolysis of ATP to ADP and lactate release in HCT116 and SW620 CRC cells were determined. Expectedly, IMP2 overexpression significantly enhanced the release of lactate and ATP contents. However, IMP2 knockdown led to a dramatically reduced release of lactate and ATP contents, and ZFAS1 could completely reverse the effects of IMP2 (Fig. 6e, f, Additional file 2: Fig. S6a, b). Cellular energy metabolism detection further demonstrated that IMP2 silencing weakened the cells’ glycolytic capacity, whereas ZFAS1 could reverse the effect of IMP2 silencing on the reduced glycolysis levels in these two types of CRC cells (Fig. 6g, h). Altogether, these findings revealed a critical connection of IMP2 stabilizing ZFAS1 expression in an m^6^A-dependent manner with OLA1-mediated mitochondrial energy metabolisms such as ATP hydrolysis and glycolysis during the occurrence and progression of CRC.

### *IMP2 promotes cell proliferation by stabilizing ZFAS1 *in vivo

To confirm the biological function of the IMP2-ZFAS1-OLA1 signal axis in vivo, a xenograft model was established by subcutaneous inoculation of four types of stably co-transfected HCT116 cells (5 × 10^6^ cells) containing groups of *shNC*, *sh*IMP2, *sh*IMP2 + *pcDH*, and *sh*IMP2 + *ZFAS1* into the armpits of 4-week-old BALB/c nude mice (Fig. 7a). In this study, a noticeable xenograft tumor appeared 10 days after transplantation, the mice were then sacrificed, and the xenograft tumor was removed on day 40 or followed until death (Fig. 7a). Specifically, the prognostic evaluation revealed that xenograft mice had a significantly shortened survival time after co-transfection with the *sh*IMP2 and *ZFAS1* vectors (Fig. 7b), suggesting the synergistic interaction of IMP2 and ZFAS1 in the CRC prognostic evaluation. Furthermore, the tumor volume, size, and weight were significantly reduced in xenograft mice transfected with the *sh*IMP2 vector; however, a dramatic increase was observed after co-transfection with the *sh*IMP2 and *ZFAS1* vectors (Fig. 7c–e). The results indicated that ZFAS1 overexpression significantly reversed the inhibition of tumor growth caused by IMP2 silencing in the xenograft mouse model. Consistently, the representative bioluminescence of xenografts collected on days 0, 10, and 40 reached the same conclusion (Fig. 7f). Furthermore, ISH and IHC assays confirmed that ZFAS1 and OLA1 expressions in the *sh*IMP2 group and *sh*IMP2 + *pcDH* group were significantly decreased compared with those in the NC group in the xenograft tumors; however, no significant difference was found between the *sh*IMP2 + *ZFAS1* group and the NC group (Fig. 7g). Consistently, qPCR and western blot assays also presented a similar alteration of these indicators, including ZFAS1, and OLA1 after co-transfection with *sh*IMP2 and *ZFAS1* vectors in the xenograft tumors (Fig. 7 h–j). Collectively, in vivo experiments demonstrated that IMP2 knockdown inhibits the tumor growth and prognosis of the xenografts via the interaction of IMP2 with the ZFAS1/OLA1 axis through m^6^A-induced stability in CRC.

## Discussion

Recently, emerging evidence supported that m^6^A modification affected various processes of tumor initiation and progression by regulating RNA stability and expression of potential targets including oncogenes and suppressor genes [24–26]. This dynamic m^6^A event is collaboratively controlled by its regulatory multicomponent methyltransferase proteins (m^6^A writers), removed by its demethylase complex proteins (m^6^A erasers), and interpreted by its modifications (m^6^A readers) within the cellular life cycle [27]. Dysregulated m^6^A readers such as the IMP family can be functionally regulated by multiple RNAs (mRNA, lncRNAs, miRNAs, etc.); however, the underlying regulatory mechanisms of m^6^A through its reader modulators involved in tumor initiation and pathogenesis remain unclear, particularly in CRC.

**Fig. 8:**
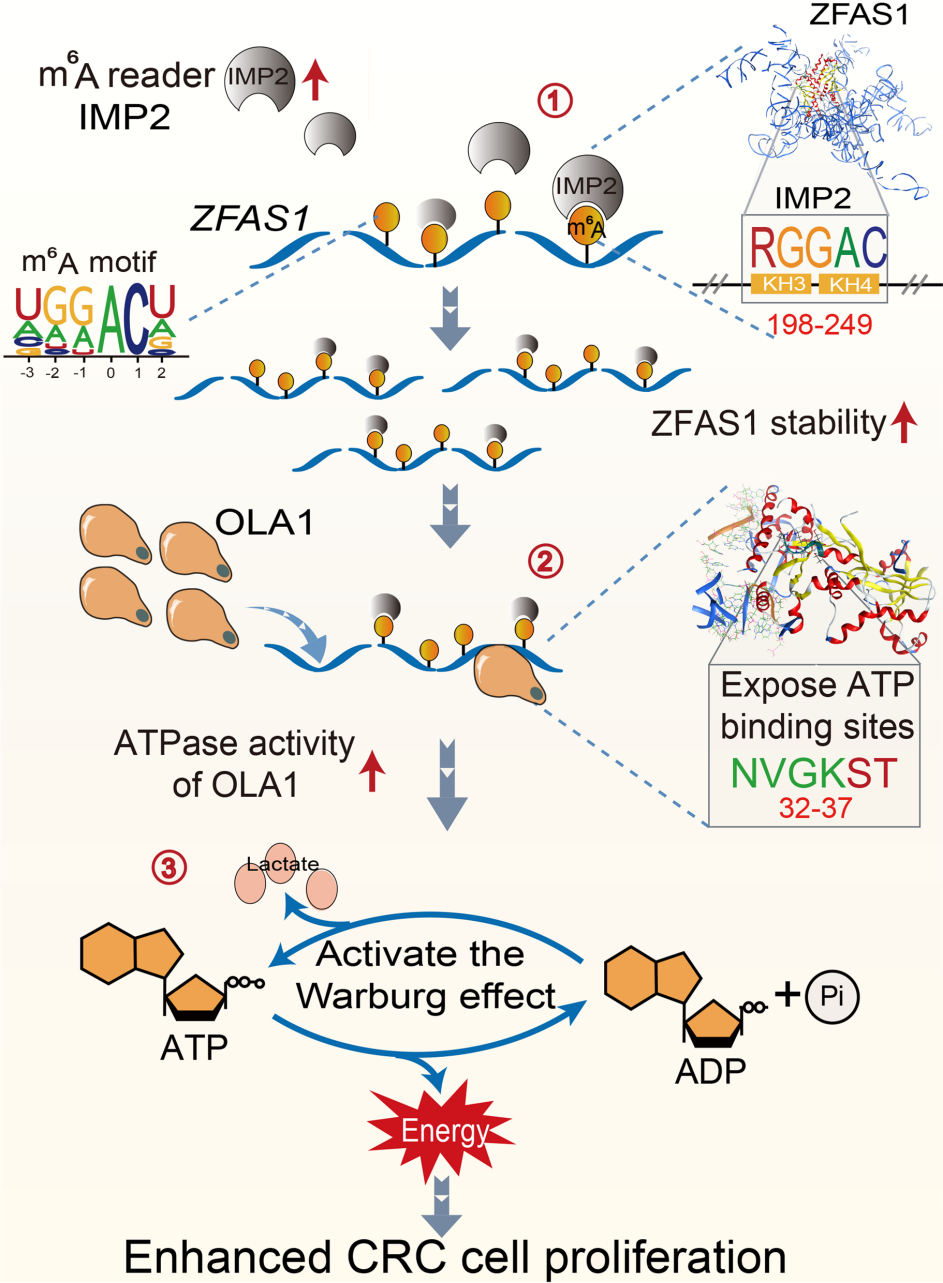
IMP2 stabilized *ZFAS1* promotes the progression of CRC by promoting the ATPase activity of OLA1. **①** The critical KH3-4 domain of m^6^A modulator IMP2 directly recognized the specific m^6^A motif (“RGGAC/RRACH”) of *ZFAS1*, promoted its stability and expression in an m^6^A dependent manner. **②** The IMP2 stabilized *ZFAS1* subsequently directly bound to the OBG-type functional domain of the OLA1 protein to fully expose the ATP binding site of the OLA1 protein, and ultimately enhanced the ATPase activity of OLA1 protein. **③** The *ZFAS1* activated OLA1 enhanced ATP-hydrolysis capacity and activated Warburg effect and ultimately promoted the progression of CRC

Altered levels of the m^6^A reader IMP1/2/3 family were reported to affect RNA metabolism processing, translation, or degradation, thereby disrupting gene stability and/or expression and critical cellular signaling bioprocesses, ultimately resulting in tumorigenesis and progression [10]. In our work, we discovered a crucial regulation network among m^6^A readers IMP2 and modified ZFAS1 by m^6^A modulation pattern involved in OLA1-mediated ATP hydrolysis and cellular glycolysis metabolism during CRC tumorigenesis and progression (Fig. 8, graphical abstract). In human CRC cells, IMP2 overexpression was accompanied by elevated m^6^A modification and ZFAS1 expression, resulting in cell proliferative promotion and cellular apoptotic inhibition by stabilizing m^6^A-modified ZFAS1. In human CRC tissues (*n* = 144), higher expressions of IMP2, ZFAS1, and m^6^A appeared to be higher risk predictors of CRC prognostic evaluation values. Consistently, Hu et al. [16] demonstrated that IMP2 inhibition suppressed cell proliferation and that high IMP2 expression was associated with worse clinical outcomes in patients with pancreatic cancer. Recently, Deng et al. [28] also assessed the prognostic values of IMP2 in head and neck squamous cell carcinoma (HNSCC) based on the TCGA dataset and patient samples, which exhibited that IMP2 serves as a potentially high-risk prognostic factor for HNSCC. To the best of our knowledge, we are the first to reveal that lncRNA ZFAS1 is positively correlated with IMP2, a master oncogenic m^6^A modulator, exhibiting a remarkable effect on cell biological characteristics and prognostic outcomes in CRC. Thus, our findings provided a series of novel biomarkers and targets in terms of epigenetic modification for CRC diagnostic prediction and clinical outcome evaluation.

Accumulating studies have provided evidence that lncRNAs performed critical functions by regulating or by being regulated through multiple m^6^A-associated writers, erasers, and readers in various cancers [7, 29–31]. Our previous publication demonstrated an LNC942–METTL14–CXCR4/

CYP1B1 signaling axis through m^6^A-dependent regulation in breast cancer cells and tissues. LNC942 was found to interact with the m^6^A regulator METTL14 to facilitate the molecular function of breast cancer cells [19]. Recently, DANCR was identified as a novel target of IMP2 through m^6^A modification, and IMP2 and DANCR exert their functions jointly to promote cancer stemness-like properties and pancreatic cancer pathogenesis [16]. In the present study, IMP2 served as an m^6^A reader to modify and stabilize ZFAS1 expression and decay. Mechanistically, ZFAS1 is recognized and modified at the + 843 site of adenosine within the “RGGAC/RRACH” motif, which is crucial for the IMP2 KH3–4 domain directly interacting with the modified ZFAS1. Despite the emerging data regarding the functions of ZFAS1 in various cancers [22, 32, 33], the epigenetic regulation network or function of an m^6^A reader such as the IMP1/2/3 family has not been reported, especially in CRC. For the first time, our work demonstrated that ZFAS1 was a novel target for IMP2 through m^6^A modification, and IMP2 and ZFAS1 jointly promoted CRC cell proliferation and pathogenesis.

The main energy metabolisms in tumors are elevated glycolysis activation and increased lactate fermentation [34]. Recently, emerging evidence revealed that m^6^A can positively regulate the glycolysis of tumor cells [7, 35, 36]. Li et al. found that m^6^A-modified pyruvate dehydrogenase kinase 4 positively regulated its translation elongation and mRNA stability by binding with the YTHDF1/eEF-2 complex and IMP3, respectively [35]. In our study, ZFAS1 overexpression caused by IMP2-dependent m^6^A stability pattern significantly enhanced the ATPase activity of OLA1, triggering with mitochondrial energy transformation including ATP hydrolysis and glycolysis. Similarly, Shen et al. found that METTL3 directly stabilized HK2 and SLC2A1 (GLUT1) and activated the glycolysis pathway, and the stabilization of these two genes was dependent on the m^6^A reader IMP2 or IMP2/3, respectively [36]. Dysregulated OLA1 has been observed in various types of human malignant tumors including lung cancer, gastric cancer, oral cancer, and CRC [37–39]. Overexpressed OLA1 plays essential roles in various bioprocesses and pathogeneses such as autophagy in the tumor cell–matrix mitochondria, oxidative stress, and release of ketones, lactic acid, and glutamine [40]. Importantly, our findings revealed a novel connection of stabilized ZFAS1 regulated by IMP2 m^6^A-recognizing manner with OLA1-associated ATP hydrolysis and glycolysis during CRC occurrence and progression. Strikingly, OLA1 expression positively correlated with IMP2 and ZFAS1 in CRC cells and tissues, which indicated that these indicators may be potential diagnostic and novel therapeutic strategies for CRC treatment.

## Conclusions

In summary, our work reveals a novel regulation mechanism by the IMP2–ZFAS1–OLA1 signal axis in CRC tumorigenesis and progression. In CRC cells, IMP2 positively regulates lncRNA ZFAS1. Ectopic IMP2 enhances, whereas knockdown IMP2 suppresses, the stability and expression of ZFAS1. Based on the RNA recognition motif, the KH3–4 domains of IMP2 protein are functionally identified as the direct RNA-binding domains contributing to RNA recognition with the + 843 m^6^A sites (“RGGAC/RRACH”) within ZFAS1. Moreover, ZFAS1 directly binds to OLA1 to enhance the ATPase activity of OLA1, which mediates mitochondrial energy metabolism including bioprocesses of ATP hydrolysis and glycolysis and ultimately affects CRC cell fate. Overall, a novel signaling axis is uncovered that identifies new targets and crosstalk involving m^6^A epigenetic modification and mitochondrial energy transformation in CRC prevention and treatment.

## Supplementary Information

**Additional file 1: Table S1.** Data of lncRNAs cluster in CRC (GSE90639). **Table S2.** Data of mRNAs and lncRNAs cluster in CRC (GSE41657). **Table S3.** Data of mRNAs and lncRNAs cluster in CRC (TCGA). **Table S4.** Data of ZFAS1-binding proteins in StarBase. **Table S5.** Correlation between IMP2 expression and clinicopathological features in the included colorectal cancer patients (n=144). **Table S6.** Correlation between m^6^A expression and clinicopathological features in the included colorectal cancer patients (n=144). **Table S7.** Correlation between ZFAS1 expression and clinicopathological features in the included colorectal cancer patients (n=144). **Table S8.** Data of mRNAs cluster of target genes in Heat map analysis (GSE137511). **Table S9.** Correlation between OLA1 expression and clinicopathological features in the included colorectal cancer patients (n=144). **Table S10.**Short hairpin RNAs (shRNAs) sequence against ZFAS1. **Table S11.** Short hairpin RNAs (shRNAs) sequence against IMP2. **Table S12.**Reverse transcription polymerase chain reaction (RT-qPCR) assays. **Table S13.** Primers used in RT-qPCR assays. **Table S14.** Probes used in situ hybridization (ISH) assay. **Table S15.** Probes used in pull down assay.

**Additional file 2: Figure S1.** The relationship of IMP1/2/3 with lncRNAs expression and clinicopathological features. **Figure S2.** Identification of the direct interaction between IMP2 and *ZFAS1* in CRC cells. **Figure S3.** IMP2 mediates biological characteristics by regulating *ZFAS1* expression in CRC cells. **Figure S4.** The relationship of ZFAS1 with OLA1 expression and clinicopathological features. **Figure S5.** Identification of the impact of *ZFAS1*-OLA1 axis on energy metabolism. **Figure S6.** Identification of the impact of IMP2-*ZFAS1*-OLA1 axis on energy metabolism.

## Data Availability

The authors declare that all the data supporting the findings in this study are available in this study and its Supplementary materials, or are available from the corresponding author through reasonable request. The Raw data were download from the Gene Expression Omnibus (GEO) website, show as follows: GSE41657 (https://www.ncbi.nlm.nih.gov/geo/query/acc.cgi?acc=GSE41657). GSE90639 (https://www.ncbi.nlm.nih.gov/geo/query/acc.cgi?acc=GSE90639). GSE137511 (https://www.ncbi.nlm.nih.gov/geo/query/acc.cgi?acc=GSE137511). GSE128435 (https://www.ncbi.nlm.nih.gov/geo/query/acc.cgi?acc=GSE128435).

## Abbreviations

IMP1/2/3: Insulin-like growth factor 2 mRNA-binding protein 1/2/3
m^6^A: N6-methyladenine
CRC: Colorectal cancer
ATP: Adenosine triphosphate
LD: Lactic acid
lncRNAs: Long non-coding RNAs
ZFAS1: ZNFX1 antisense RNA 1
OLA1: Obg-like ATPase 1
TMA: Tissue microarray
IHC: Immunohistochemistry
RIP: RNA-binding protein immunoprecipitation assay
CFA: Colony-forming assays
IF: Immunofluorescence
ISH: In situ hybridization
ROC curve: Receiver operating characteristic curve
DFS: Disease-free survival
OS: Overall survival
TCGA: The Cancer Genome Atlas
HIEC: Human intestinal epithelial cells
