# Supplementary material for: N6-methyladenosine reader IMP2 stabilizes the ZFAS1/OLA1 axis and activates the Warburg effect: implication in colorectal cancer
Source: J Hematol Oncol. 2021 Nov 7;14:188. doi: 10.1186/s13045-021-01204-0 (PMC8574039; doi:10.1186/s13045-021-01204-0)
Supplement: Supplementary file 1 — Additional file 1: Table S1. Data of lncRNAs cluster in CRC (GSE90639). Table S2. Data of mRNAs and lncRNAs cluster in CRC (GSE41657). Table S3. Data of mRNAs and lncRNAs cluster in CRC (TCGA). Table S4. Data of ZFAS1-binding proteins in StarBase. Table S5. Correlation between IMP2 expression and clinicopathological features in the included colorectal cancer patients (n=144). Table S6. Correlation between m6A expression and clinicopathological features in the included colorectal cancer patients (n=144). Table S7. Correlation between ZFAS1 expression and clinicopathological features in the included colorectal cancer patients (n=144). Table S8. Data of mRNAs cluster of target genes in Heat map analysis (GSE137511). Table S9. Correlation between OLA1 expression and clinicopathological features in the included colorectal cancer patients (n=144). Table S10.Short hairpin RNAs (shRNAs) sequence against ZFAS1. Table S11. Short hairpin RNAs (shRNAs) sequence against IMP2. Table S12.Reverse transcription polymerase chain reaction (RT-qPCR) assays. Table S13. Primers used in RT-qPCR assays. Table S14. Probes used in situ hybridization (ISH) assay. Table S15. Probes used in pull down assay. [file 13045_2021_1204_MOESM1_ESM.docx]

**Additional file 1**

**N6-methyladenosine Reader IMP2 Stabilizes the ZFAS1/OLA1 Axis and Activates the Warburg Effect: Implication in Colorectal Cancer**

**This file includes:**

- **Table S1.** Data of lncRNAs cluster in CRC (GSE90639)
- **Table S2.** Data of mRNAs and lncRNAs cluster in CRC (GSE41657)
- **Table S3.** Data of mRNAs and lncRNAs cluster in CRC (TCGA)
- **Table S4.** Data of ZFAS1-binding proteins in StarBase
- **Table S5.** Correlation between IMP2 expression and clinicopathological features in the included colorectal cancer patients (*n*=144)
- **Table S6.** Correlation between m^6^A expression and clinicopathological features in the included colorectal cancer patients (*n*=144)
- **Table S7.** Correlation between ZFAS1 expression and clinicopathological features in the included colorectal cancer patients (*n*=144)
- **Table S8.** Data of mRNAs cluster of target genes in Heat map analysis (GSE137511)
- **Table S9.** Correlation between OLA1 expression and clinicopathological features in the included colorectal cancer patients (*n*=144)
- **Table S10.** *Short hairpin RNAs (shRNAs)* sequence against ZFAS1
- **Table S11.** *Short hairpin RNAs (shRNAs)* sequence against IMP2
- **Table S12.** Reverse transcription polymerase chain reaction (RT-qPCR) assays
- **Table S13.** Primers used in RT-qPCR assays
- **Table S14.** Probes used in situ hybridization (ISH) assay
- **Table S15.** Probes used in pull down assay

**Table S1. Data of lncRNAs cluster in CRC (GSE90639)**

| **Fold Change** | | | |
| --- | --- | --- | --- |
| **LncRNA** | **IGF2BP1** | **IGF2BP2** | **IGF2BP3** |
| \| CRNDE \| \| --- \| \| SNHG15 \| \| KIFC1 \| \| CABIN1 \| \| **ZFAS1** \| \| HCG18 \| \| SNHG7 \| | \| 2.95 \| \| --- \| \| 4.67 \| \| 3.24 \| \| 1.90 \| \| 2.99 \| \| 2.23 \| \| 3.81 \| | \| 4.62 \| \| --- \| \| 5.34 \| \| 3.53 \| \| 1.83 \| \| **4.59** \| \| 1.78 \| \| 3.80 \| | \| 4.35 \| \| --- \| \| 5.04 \| \| 5.33 \| \| 2.58 \| \| 4.29 \| \| 2.46 \| \| 5.14 \| |

**Table S2. Data of mRNAs and lncRNAs cluster in CRC (GSE41657)**

| **Up-regulated** | | **Up-regulated** | |
| --- | --- | --- | --- |
| **lncRNA** | **Fold Change** | **mRNA** | **Fold Change** |
| \| CRNDE \| \| --- \| \| SNHG15 \| \| KIFC1 \| \| CABIN1 \| \| **ZFAS1** \| \| HCG18 \| \| SNHG7 \| | \| 5.86 \| \| --- \| \| 3.75 \| \| 3.55 \| \| 2.69 \| \| **2.50** \| \| 2.33 \| \| 2.21 \| | \| IMP1 \| \| --- \| \| **IMP2** \| \| IMP3 \| | \| 1.54 \| \| --- \| \| **1.58** \| \| 3.13 \| |

**Table S3. Data of mRNAs and lncRNAs cluster in CRC (TCGA)**

| **Up-regulated** | | **Up-regulated** | |
| --- | --- | --- | --- |
| **lncRNA** | **logFC** | **mRNA** | **logFC** |
| \| **ZFAS1** \| \| --- \| \| KIFC1 \| \| SNHG7 \| \| SNHG15 \| \| CRNDE \| \| CABIN1 \| \| HCG18 \| | \| **121.08** \| \| --- \| \| 26.25 \| \| 18.01 \| \| 17.56 \| \| 5.76 \| \| 4.59 \| \| 2.71 \| | \| IMP1 \| \| --- \| \| **IMP2** \| \| IMP3 \| | \| 1.90 \| \| --- \| \| **14.16** \| \| 1.85 \| |

**Table S4. Data of ZFAS1-binding proteins in StarBase**

| **Protein name** | **ClipSiteNum** |
| --- | --- |
| \| U2AF2 \| \| --- \| \| ELAVL1 \| \| **IGF2BP2** \| \| HNRNPC \| \| RBM10 \| \| SRSF1 \| \| UPF1 \| \| NOP58 \| \| DDX54 \| \| RBFOX2 \| \| FBL \| \| HNRNPA1 \| \| EIF4A3 \| \| CSTF2T \| \| IGF2BP3 \| \| FMR1 \| \| SRSF7 \| \| IGF2BP1 \| \| HNRNPM \| \| NOP56 \| | \| 146 \| \| --- \| \| 127 \| \| **111** \| \| 93 \| \| 80 \| \| 72 \| \| 65 \| \| 59 \| \| 57 \| \| 45 \| \| 45 \| \| 44 \| \| 40 \| \| 36 \| \| 35 \| \| 33 \| \| 29 \| \| 28 \| \| 28 \| \| 25 \| |

**Table S5. Correlation between IMP2 expression and clinicopathological features in the included colorectal cancer patients (*n* = 144)**

| **Characteristics** | ***n* =144** | **IMP2 expression** | | ***P*-value** | **Adjusted OR(95%CI)** |
| --- | --- | --- | --- | --- | --- |
|  |  | **Low (%)**  **n=52** | **High (%)**  ***n*=92** |  |  |
| **Age** |  |  |  |  |  |
| <=64 | 70 | 25(35.7) | 45(64.3) | 1.000 |  |
| >64 | 74 | 27(36.5) | 47(63.5) | 0.680 | 0.858(0.414-1.779) |
| **Gender** |  |  |  |  |  |
| Male | 82 | 36(43.9) | 46(56.1) | 0.035 |  |
| Female | 62 | 16(25.8) | 46(74.2) | 0.018 | 2.511(1.173-5.373) |
| **Family History** |  |  |  |  |  |
| No | 131 | 46(35.1) | 85(64.9) | 0.547 |  |
| Yes | 13 | 6(46.2) | 7(53.8) | 0.221 | 2.122(0.636-7.082) |
| **Differentiated Degree** |  |  |  |  |  |
| Low | 77 | 31(40.3) | 46(59.7) | 0.446 |  |
| Medium | 57 | 17(29.8) | 40(70.2) | 0.587 | 0.676(0.165-2.775) |
| High | 10 | 4(40.0) | 6(60.0) | 0.339 | 0.496(0.118-2.086) |
| **Primary Organ** |  |  |  |  |  |
| Colon | 70 | 24(34.3) | 46(65.7) | 0.729 |  |
| Rectum | 74 | 28(37.8) | 46(62.2) | 0.409 | 0.736(0.356-1.522) |
| **Infiltration** |  |  |  |  |  |
| Negative | 117 | 43(36.8) | 74(63.2) | 0.826 |  |
| Positive | 27 | 9(33.3) | 18(66.7) | 0.717 | 1.178(0.485-2.860) |
| **Size** |  |  |  |  |  |
| <=6 | 77 | 30(39.0) | 47(61.0) | 0.489 |  |
| >6 | 67 | 22(32.8) | 45(67.2) | 0.175 | 1.674(0.795-3.523) |
| **DFS** |  |  |  |  |  |
| 0 = progression | 46 | 23(50.0) | 23(50.0) | **0.025** |  |
| 1 = death | 98 | 29(29.6) | 69(70.4) | **0.007** | 0.339(0.155-0.739) |
| **OS** |  |  |  |  |  |
| 0 = alive | 53 | 26(49.1) | 27(50.9) | **0.019** |  |
| 1 = death | 91 | 26(28.6) | 65(71.4) | **0.007** | 0.362(0.173-0.757) |

Abbreviations: 95%CI, 95% confidence interval; DFS, Disease-free survival; OS, Overall survival.

*P* values, Adjusted HR (95%CI) were assessed using *Pearson* *χ*^2^ test and logistic regression analysis adjusted for age, differentiation.

**Table S6. Correlation between m^6^A expression and clinicopathological features in the included colorectal cancer patients (*n* = 144)**

| **Characteristics** | ***n* =144** | **m^6^A expression** | | ***P*-value** | **Adjusted OR(95%CI)** |
| --- | --- | --- | --- | --- | --- |
|  |  | **Low (%)**  **n=89** | **High (%)**  ***n*=55** |  |  |
| **Age** |  |  |  |  |  |
| <=64 | 70 | 41(58.6) | 29(41.4) | 0.494 |  |
| >64 | 74 | 48(64.9) | 26(35.1) | 0.317 | 1.430(0.710-2.883) |
| **Gender** |  |  |  |  |  |
| Male | 82 | 53(64.6) | 29(35.4) | 0.489 |  |
| Female | 62 | 36(58.1) | 26(41.9) | 0.121 | 0.548(0.256-1.173) |
| **Family History** |  |  |  |  |  |
| No | 131 | 81(61.8) | 50(38.2) | 1.000 |  |
| Yes | 13 | 8(61.5) | 5(38.5) | 0.737 | 0.814(0.244-2.710) |
| **Differentiated Degree** |  |  |  |  |  |
| Low | 77 | 53(68.8) | 24(31.2) | 0.032 |  |
| Medium | 57 | 28(49.1) | 29(50.9) | 0.219 | 2.948(0.526-16.524) |
| High | 10 | 8(80.0) | 2(20.0) | 0.040 | 6.153(1.085-34.886) |
| **Primary Organ** |  |  |  |  |  |
| Colon | 70 | 52(74.3) | 18(25.7) | 0.003 |  |
| Rectum | 74 | 37(50.0) | 37(50.0) | 0.002 | 0.272(0.118-0.631) |
| **Infiltration** |  |  |  |  |  |
| Negative | 117 | 72(61.5) | 45(38.5) | 1.000 |  |
| Positive | 27 | 17(63.0) | 10(37.0) | 0.611 | 1.266(0.509-3.148) |
| **Size** |  |  |  |  |  |
| <=6 | 77 | 45(58.4) | 32(41.6) | 0.395 |  |
| >6 | 67 | 44(65.7) | 23(34.3) | 0.374 | 1.360(0.691-2.680) |
| **DFS** |  |  |  |  |  |
| 0 = progression | 46 | 35(76.1) | 11(23.9) | **0.017** |  |
| 1 = death | 98 | 54(55.1) | 44(44.9) | **0.015** | 2.690(1.212-5.968) |
| **OS** |  |  |  |  |  |
| 0 = alive | 53 | 40(75.5) | 13(24.5) | **0.013** |  |
| 1 = death | 91 | 49(53.8) | 42(46.2) | **0.009** | 2.784(1.297-5.974) |

Abbreviations: 95%CI, 95% confidence interval; DFS, Disease-free survival; OS, Overall survival.

*P* values, Adjusted HR (95%CI) were assessed using *Pearson* *χ*^2^ test and logistic regression analysis adjusted for age, differentiation.

**Table S7. Correlation between ZFAS1 expression and clinicopathological features in the included colorectal cancer patients (*n* = 144)**

| **Characteristics** | ***n* =144** | **ZFAS1 expression** | | ***P*-value** | **Adjusted OR(95%CI)** |
| --- | --- | --- | --- | --- | --- |
|  |  | **Low (%)**  **n=100** | **High (%)**  ***n*=44** |  |  |
| **Age** |  |  |  |  |  |
| <=64 | 70 | 49(70.0) | 21(30.0) | 1.000 |  |
| >64 | 74 | 51(68.9) | 23(31.1) | 0.886 | 0.949(0.467-1.931) |
| **Gender** |  |  |  |  |  |
| Male | 82 | 65(79.3) | 17(20.7) | 0.004 |  |
| Female | 62 | 35(56.5) | 27(43.5) | 0.001 | 0.271(0.122-0.606) |
| **Family History** |  |  |  |  |  |
| No | 131 | 92(70.2) | 39(29.8) | 0.537 |  |
| Yes | 13 | 8(61.5) | 5(38.5) | 0.447 | 1.606(0.473-5.453) |
| **Differentiated Degree** |  |  |  |  |  |
| Low | 77 | 54(70.1) | 23(29.9) | 0.798 |  |
| Medium | 57 | 40(70.2) | 17(29.8) | 0.477 | 0.584(0.133-2.571) |
| High | 10 | 6(60.0) | 4(40.0) | 0.426 | 0.540(0.119-2.462) |
| **Primary Organ** |  |  |  |  |  |
| Colon | 70 | 47(67.1) | 23(32.9) | 0.591 |  |
| Rectum | 74 | 53(71.6) | 21(28.4) | 0.417 | 1.360(0.648-2.855) |
| **Infiltration** |  |  |  |  |  |
| Negative | 117 | 84(71.8) | 33(28.2) | 0.247 |  |
| Positive | 27 | 16(59.3) | 11(40.7) | 0.205 | 0.571(0.240-1.358) |
| **Size** |  |  |  |  |  |
| <=6 | 77 | 53(68.8) | 24(31.2) | 1.000 |  |
| >6 | 67 | 47(70.1) | 20(29.9) | 0.659 | 0.844(0.397-1.793) |
| **DFS** |  |  |  |  |  |
| 0 = progression | 46 | 40(87.0) | 6(13.0) | **0.002** |  |
| 1 = death | 98 | 60(61.2) | 38(33.8) | **0.002** | 4.670(1.738-12.548) |
| **OS** |  |  |  |  |  |
| 0 = alive | 53 | 43(81.1) | 10(18.9) | **0.024** |  |
| 1 = death | 91 | 57(62.6) | 34(37.4) | **0.022** | 2.575(1.146-5.787) |

Abbreviations: 95%CI, 95% confidence interval; DFS, Disease-free survival; OS, Overall survival.

*P* values, Adjusted HR (95%CI) were assessed using *Pearson* *χ*^2^ test and logistic regression analysis adjusted for age, differentiation.

**Table S8. Data of mRNAs cluster of target genes in Heat map analysis (GSE137511)**

| **mRNA** | **Fold Change** | **mRNA** | **Fold Change** |
| --- | --- | --- | --- |
| \| **OLA1** \| \| --- \| \| TP53RK \| \| RPS27A \| \| AHCY \| \| RPL39 \| \| ZC3H8 \| \| RPS2 \| \| RPL29 \| \| RPL18 \| \| NME2 \| \| DPM1 \| \| RPL7 \| \| CCT4 \| \| CCT7 \| \| NOB1 \| \| RPS3 \| \| RPS25 \| \| ZCCHC7 \| \| RPL8 \| \| IGBP1 \| \| EIF2S2 \| \| RBMX \| \| BTF3 \| \| RPS10 \| | \| **2.25** \| \| --- \| \| 2.02 \| \| 1.68 \| \| 2.88 \| \| 3.55 \| \| 2.08 \| \| 2.66 \| \| 2.38 \| \| 1.62 \| \| 1.57 \| \| 2.82 \| \| 2.39 \| \| 2.64 \| \| 2.47 \| \| 3.55 \| \| 2.54 \| \| 1.65 \| \| 1.85 \| \| 2.40 \| \| 2.93 \| \| 2.92 \| \| 1.85 \| \| 1.86 \| \| 1.89 \| | \| RPL19 \| \| --- \| \| RPS11 \| \| RPS13 \| \| RPL11 \| \| PPA1 \| \| RPL37A \| \| RPL28 \| \| RPL31 \| \| RPL14 \| \| RPL30 \| \| RPL37 \| \| RPS20 \| \| RPL35A \| \| EIF2A \| \| RSL1D1 \| \| RAE1 \| \| RPS7 \| \| EIF3H \| \| RPS21 \| \| PABPC1 \| \| NPM1 \| \| EIF3M \| \| RPS14 \| | \| 1.63 \| \| --- \| \| 1.81 \| \| 2.45 \| \| 1.62 \| \| 4.21 \| \| 1.92 \| \| 1.71 \| \| 1.78 \| \| 1.87 \| \| 2.18 \| \| 1.90 \| \| 2.01 \| \| 1.85 \| \| 2.01 \| \| 3.29 \| \| 1.80 \| \| 2.20 \| \| 2.20 \| \| 3.34 \| \| 1.93 \| \| 2.41 \| \| 2.52 \| \| 2.03 \| |

**Table S9. Correlation between OLA1 expression and clinicopathological features in the included colorectal cancer patients (*n* = 144)**

| **Characteristics** | ***n* =144** | **OLA1 expression** | | ***P*-value** | **Adjusted OR(95%CI)** |
| --- | --- | --- | --- | --- | --- |
|  |  | **Low (%)**  **n=105** | **High (%)**  ***n*=39** |  |  |
| **Age** |  |  |  |  |  |
| <=64 | 70 | 51(72.9) | 19(27.1) | 1.000 |  |
| >64 | 77 | 54(73.0) | 20(27.0) | 0.701 | 1.169(0.526-2.596) |
| **Gender** |  |  |  |  |  |
| Male | 82 | 60(73.2) | 22(26.8) | 1.000 |  |
| Female | 62 | 45(72.6) | 17(27.4) | 0.879 | 0.941(0.421-2.051) |
| **Family History** |  |  |  |  |  |
| No | 131 | 98(74.8) | 33(25.2) | 0.114 |  |
| Yes | 13 | 7(53.8) | 6(46.2) | 0.112 | 2.567(0.802-8.222) |
| **Differentiated Degree** |  |  |  |  |  |
| Low | 77 | 57(74.0) | 20(26.0) | 0.348 |  |
| Medium | 57 | 39(68.4) | 18(31.6) | 0.110 | 5.951(0.668-53.044) |
| High | 10 | 9(90.0) | 1(10.0) | 0.119 | 5.727(0.640-51.256) |
| **Primary Organ** |  |  |  |  |  |
| Colon | 70 | 48(68.6) | 22(31.4) | 0.267 |  |
| Rectum | 74 | 57(77.0) | 17(23.0) | 0.158 | 1.795(0.796-4.049) |
| **Infiltration** |  |  |  |  |  |
| Negative | 117 | 87(74.4) | 30(25.6) | 0.473 |  |
| Positive | 27 | 18(66.7) | 9(33.3) | 0.400 | 0.673(0.268-1.692) |
| **Size** |  |  |  |  |  |
| <=6 | 77 | 58(75.3) | 19(24.7) | 0.574 |  |
| >6 | 67 | 47(70.1) | 20(29.9) | 0.168 | 0.571(0.257-1.267) |
| **DFS** |  |  |  |  |  |
| 0 = progression | 46 | 42(91.3) | 4(8.7) | **0.001** |  |
| 1 = death | 98 | 63(64.3) | 35(35.7) | **0.001** | 7.327(2.315-23.185) |
| **OS** |  |  |  |  |  |
| 0 = alive | 53 | 47(88.7) | 6(11.3) | **0.001** |  |
| 1 = death | 91 | 58(63.7) | 33(36.3) | **0.001** | 4.7911.825-12.577) |

Abbreviations: 95%CI, 95% confidence interval; DFS, Disease-free survival; OS, Overall survival.

*P* values, Adjusted HR (95%CI) were assessed using *Pearson* *χ*^2^ test and logistic regression analysis adjusted for age, differentiation.

**Table S10. *Short hairpin RNAs (shRNAs)* sequence against ZFAS1**

| **shRNA** | **Sequence (5'-3')** |
| --- | --- |
| ZFAS1-Homo-455 | GCCATTCGTTCTTTCGCGTCT |
| ZFAS1-Homo-525 | GCTATTGTCCTGCCCGTTAGA |
| ZFAS1-Homo-769(shRNA-1) | GATTCAGTCTGCCTTGTAACA |
| ZFAS1-Homo-884(shRNA-2) | CAAGGTTACTGTATACATAGC |

**Table S11. *Short hairpin RNAs (shRNAs)* against IMP2**

| **shRNA** | **Sequence (5'-3')** |
| --- | --- |
| IGF2BP2-Homo-6350(shRNA-1) | CTTAACCAGTGCAGAAGTCAT |
| IGF2BP2-Homo-6351 | CGGATCTTTGGGAAACTGAAA |
| IGF2BP2-Homo-6352(shRNA-2) | CAGTGCTGAGATAGAGATTAT |

**Table S12. Reverse transcription polymerase chain reaction (RT-qPCR) assays**

| **mRNA and LncRNA** | |
| --- | --- |
| **Relative reagents** | **1×system (10μL)** |
| 5×RT Buffer | 2 μL |
| Enzyme Mix | 0.5 μL |
| Primer Mix | 0.5 μL |
| RNA+DEPC H_2_O | 7 μL (400ng) |
| **Program: 37℃/15min→ 98℃/5min→ Maintain at 4℃** | |
| **qPCR** | |
| **Relative reagents** | **1×system(10μL)** |
| SYBR | 5 μL |
| ROX | 0.2 μL |
| Forward Primer(10μM) | 0.4 μL |
| Reverse Primer(10μM) | 0.4 μL |
| cDNA | 4 μL |
| **Program: 95℃/60sec→[95℃/15sec→ 60℃/15sec→72℃/45sec]×40cycles→ Maintain at 4℃** | |

**Table S13. Primers used in RT-qPCR assays**

| **Primer** | **Sequence (5'-3')** |
| --- | --- |
| ZFAS1-Forward | GCTATTGTCCTGCCCGTTAG |
| ZFAS1-Reverse | TCGTCAGGAGATCGAAGGTT |
| IMP2-Forward | AATCTCTTCATCCCAACCCAG |
| IMP2-Reverse | ATGACCATCCTTTCGCTGAC |
| OLA1-Forward | CCTAATGAGAGCAGAGTACCTG |
| OLA1-Reverse | CGTGCGTGATATCATCATCTTC |
| GAPDH-Forward | CTCTGCTCCTCCTGTTCGAC |
| GAPDH-Reverse | ACCAAATCCGTTGACTCCGA |
| SNHG15-Forward | GCTGAGGTGACGGTCTCAAA |
| SNHG15-Reverse | GCCTCCCAGTTTCATGGACA |
| CRNDE-Forwar | ATATTCAGCCGTTGGTCTTTGA |
| CRNDE-Reverse | TCTGCGTGACAACTGAGGATTT |

**Table S14. Probes used in situ hybridization (ISH) assay**

| **Digoxin-labeled probe** | **Sequence (5'-3')** | |
| --- | --- | --- |
| ZFAS1-(1) | | GGAACCCGTCGAGCGGTTTGGTGCGTGTGAAGCGACAT |
| ZFAS1-(2) | | GGTTATATAAGGGAGGTTCAGGAAGCCATTCGTTCTTG |
| ZFAS1-(3) | | CTACAACCTTCGATCTCCTGACGAGTTTATTGTTGGCCAA |

**Table S15. Probes used in pull down assay**

| **Probe (ZFAS1-IMP2)** | **Sequence (5'-3')** | |
| --- | --- | --- |
| Wild type | | Bi AAACCm^6^ACACm^6^ACCCUCUCCUUUCAUCAAACUCAm^6^ACAUCU |
| Mutant type | | Bi AAACCACACACCCUCUCCUUUCAUCAAACUCAACAUCU |

| **Probe (ZFAS1-OLA1)** | **Sequence (5'-3')** | |
| --- | --- | --- |
| Wild type | | Bi AACCCUCGGUGCACAUGGUCACA |
| Mutant type | | Bi AACGGACGGUGCACAUGCACACA |
