# Supplementary material for: N6-methyladenosine reader IMP2 stabilizes the ZFAS1/OLA1 axis and activates the Warburg effect: implication in colorectal cancer
Source: J Hematol Oncol. 2021 Nov 7;14:188. doi: 10.1186/s13045-021-01204-0 (PMC8574039; doi:10.1186/s13045-021-01204-0)
Supplement: Supplementary file 2 — Additional file 2: Figure S1. The relationship of IMP1/2/3 with lncRNAs expression and clinicopathological features. Figure S2. Identification of the direct interaction between IMP2 and ZFAS1 in CRC cells. Figure S3. IMP2 mediates biological characteristics by regulating ZFAS1 expression in CRC cells. Figure S4. The relationship of ZFAS1 with OLA1 expression and clinicopathological features. Figure S5. Identification of the impact of ZFAS1-OLA1 axis on energy metabolism. Figure S6. Identification of the impact of IMP2-ZFAS1-OLA1 axis on energy metabolism. [file 13045_2021_1204_MOESM2_ESM.docx]

**Additional file 2**

**N6-methyladenosine Reader IMP2 Stabilizes the ZFAS1/OLA1 Axis and Activates the Warburg Effect: Implication in Colorectal Cancer**

## This file includes:

- **Fig. S1** The relationship of IMP1/2/3 with lncRNAs expression and clinicopathological features
- **Fig. S2** Identification of the direct interaction between IMP2 and *ZFAS1* in CRC cells
- **Fig. S3** IMP2 mediates biological characteristics by regulating *ZFAS1* expression in CRC cells
- **Fig. S4** The relationship of *ZFAS1* with OLA1 expression and clinicopathological features
- **Fig. S5** Identification of the impact of *ZFAS1*-OLA1 axis on energy metabolism
- **Fig. S6** Identification of the impact of IMP2-*ZFAS1*-OLA1 axis on energy metabolism

**
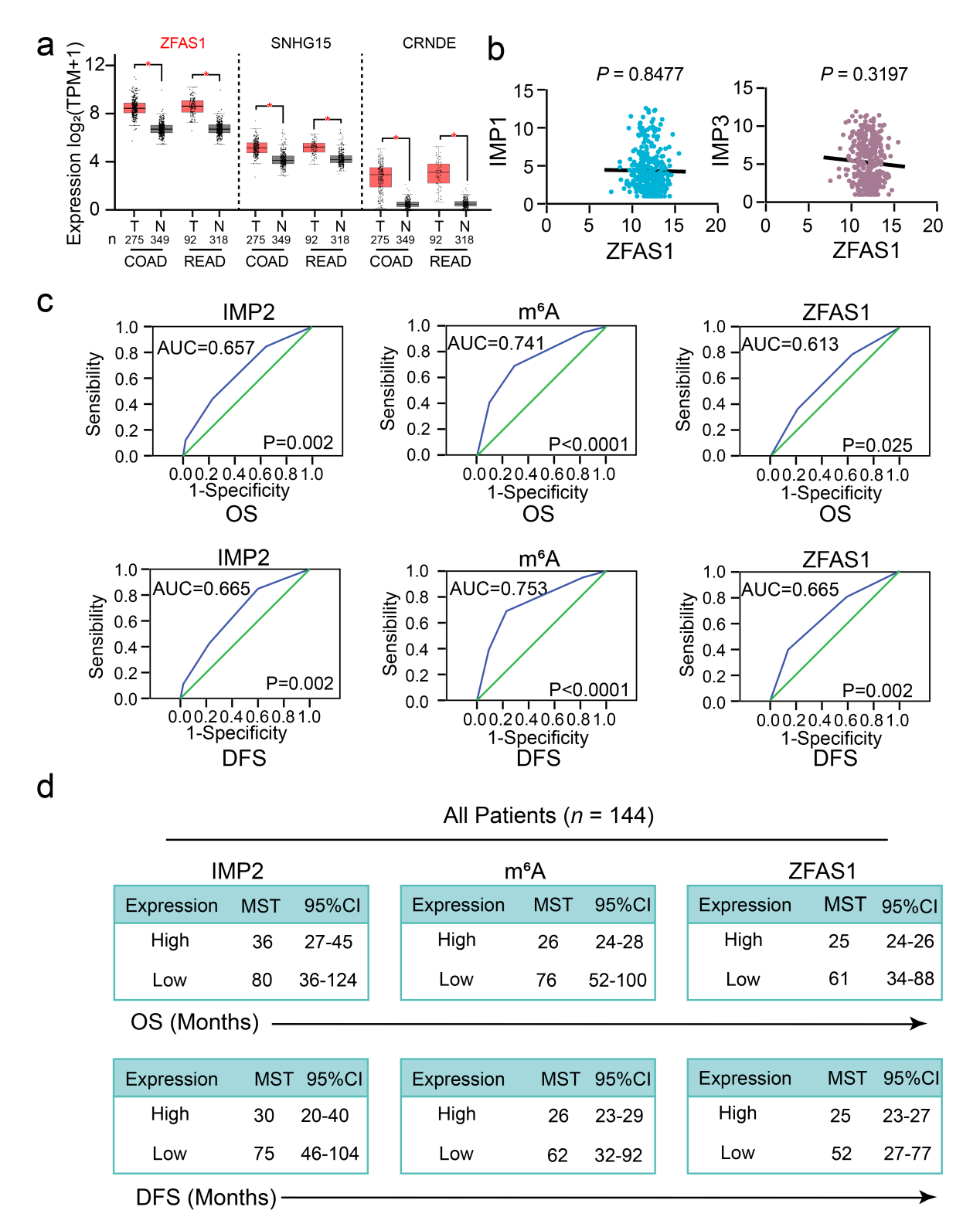
**

**Fig. S1 The relationship of IMP1/2/3 with lncRNAs expression and clinicopathological features**

**a** Expression of *ZFAS1*, *SNHG15* and *CRNDE* in COAD and READ tissues. **b** Linear correlation pattern showing a negative relationship between the expression of IMP1/3 and *ZFAS1* based on TCGA dataset. **c** The ROC curve method illustrating the cutoff values of IMP2, m^6^A and *ZFAS1* high/low expression in paired CRC patient tissues vs. adjacent-tumor controls (n=144). **d** Kaplan-Meier plot curves showing the association of IMP2 high/low expression, m^6^A high/low expression, and lncRNA *ZFAS1* high/low expression with the OS and DFS in this included CRC patients.

**
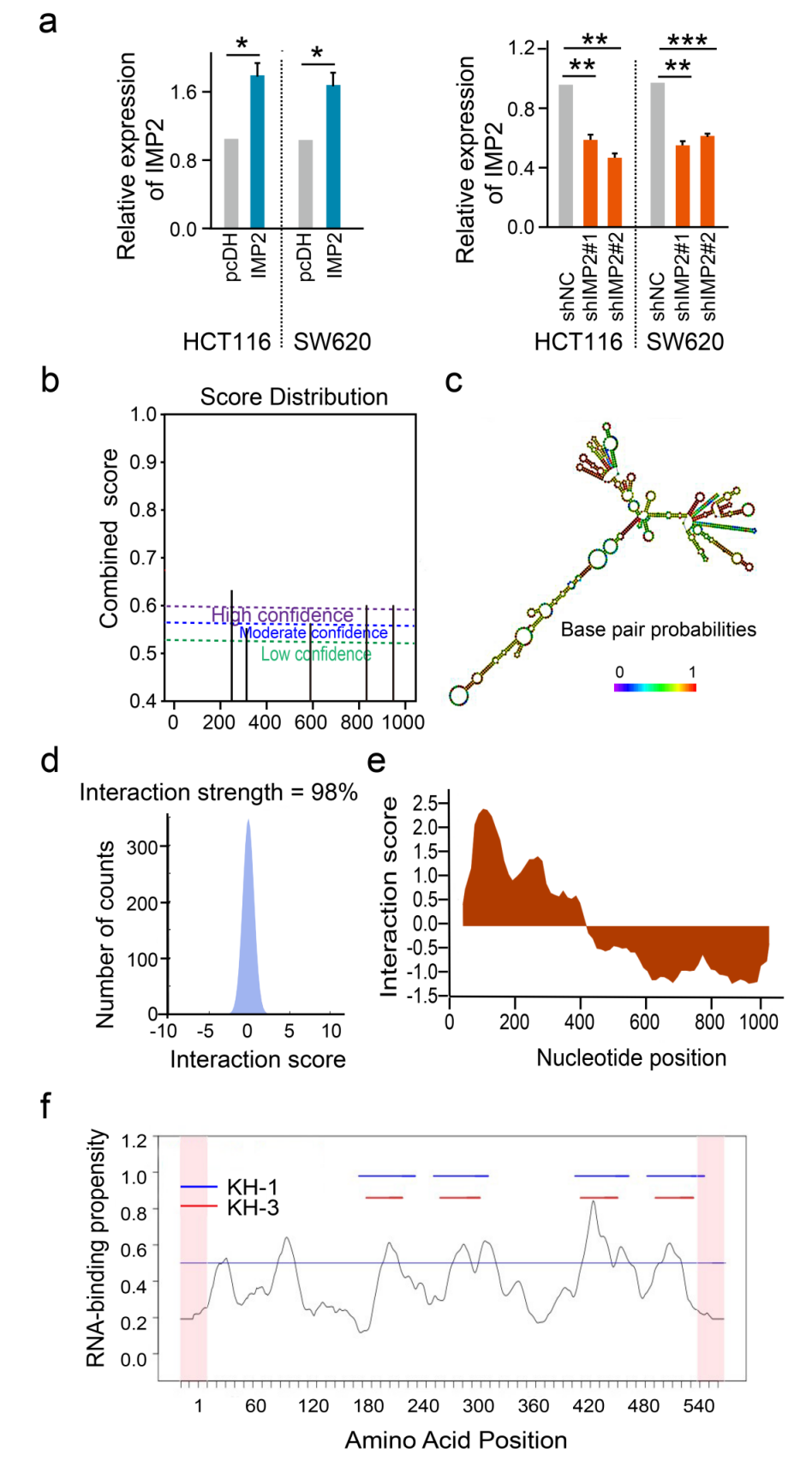
**

**Fig. S2 Identification of the direct interaction between IMP2 and *ZFAS1* in CRC cells**

**a** The expression of *IMP2* after overexpression or silencing *IMP2* in HCT116 and SW620 cells by qPCR. **b** Cuilab (http://www.cuilab.cn/) online platform showing five qualified m^6^A binding sites (RAGAC) of *ZFAS1*. **c** Predicting the secondary structure of *ZFAS1* by RNAfold (http://rna.tbi.univie.ac.at/cgi-bin/RNAWebSuite/RNAfold.cgi). **d, e, f** Bioinformatics online software predicting the interaction score of *ZFAS1* and RNA-binding propensity of IMP2 (http://www.tartaglialab.com/).

**
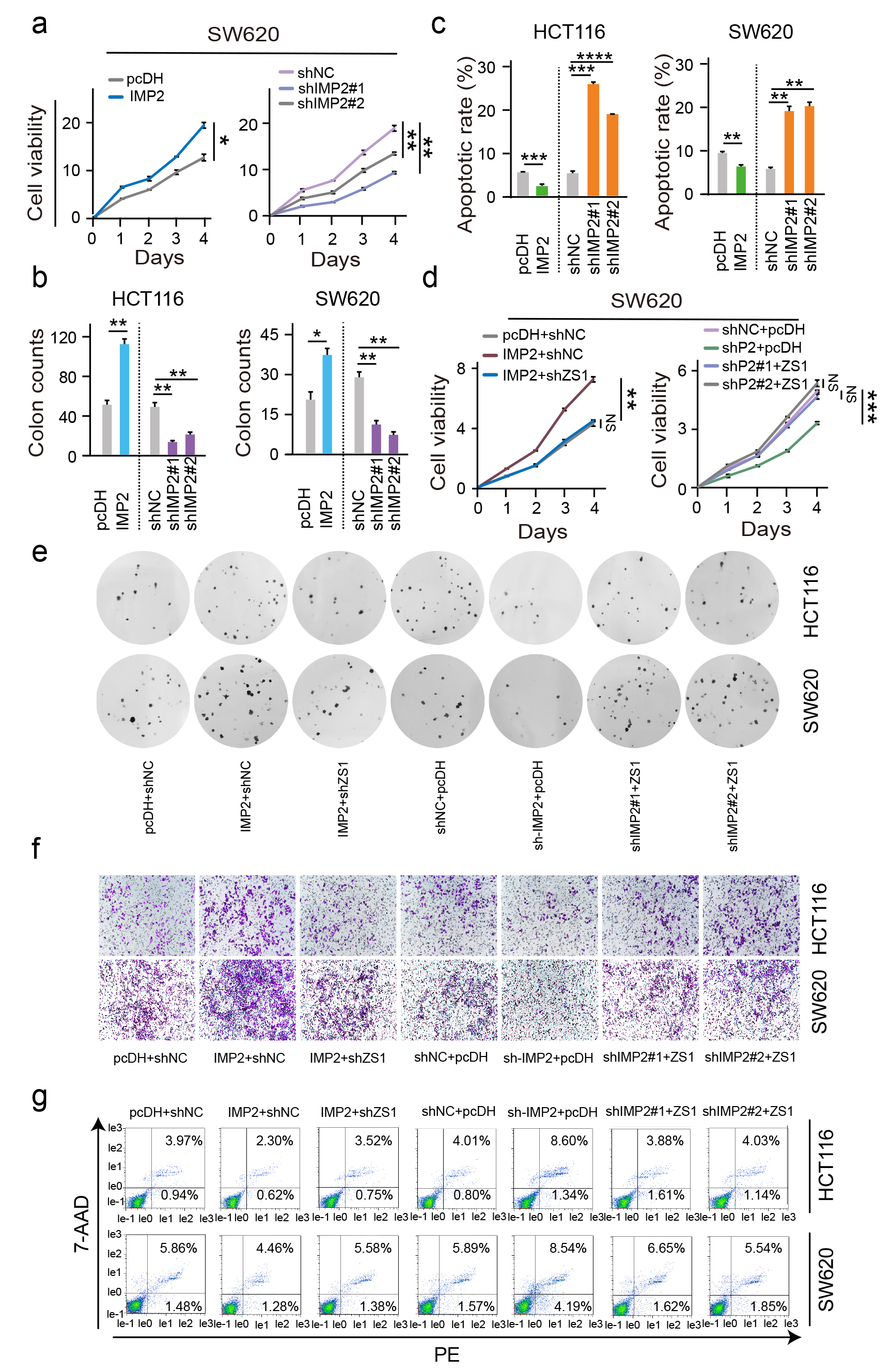
**

**Fig. S3 IMP2 mediates biological characteristics by regulating *ZFAS1* expression in CRC cells**

**a** MTT assay was performed to identify the cell viability after ectopic or silencing *IMP2* in SW620 cells. **b** CFA displaying the colony-forming abilities in both HCT116 and SW620 cells after ectopic or silencing *IMP2*. **c** The percentage (%) of cell apoptosis was detected upon *IMP2* overexpressing or silencing in HCT116 and SW620 cells by Flow cytometry. **d** Rescue experiments detecting the cell viability treated by co-transfected *shIMP2* and *ZFAS1*/*IMP2* and *shZFAS1* vectors in SW620 cells assayed by MTT assay. **e** Rescue experiments detecting the colony-forming abilities treated by co-transfected *shIMP2* and *ZFAS1*/*IMP2* and *shZFAS1* vectors in both HCT116 and SW620 cells assayed by CFA. **f** Rescue experiments detecting the migration ability treated by co-transfected *shIMP2* and *ZFAS1*/*IMP2* and *shZFAS1* vectors in both HCT116 and SW620 cells assayed by trans-well assay. **g** Rescue experiments detecting the percentage (%) of cell apoptosis treated by co-transfected *shIMP2* and *ZFAS1*/*IMP2* and *shZFAS1* vectors in both HCT116 and SW620 cells assayed by Flow cytometry.**
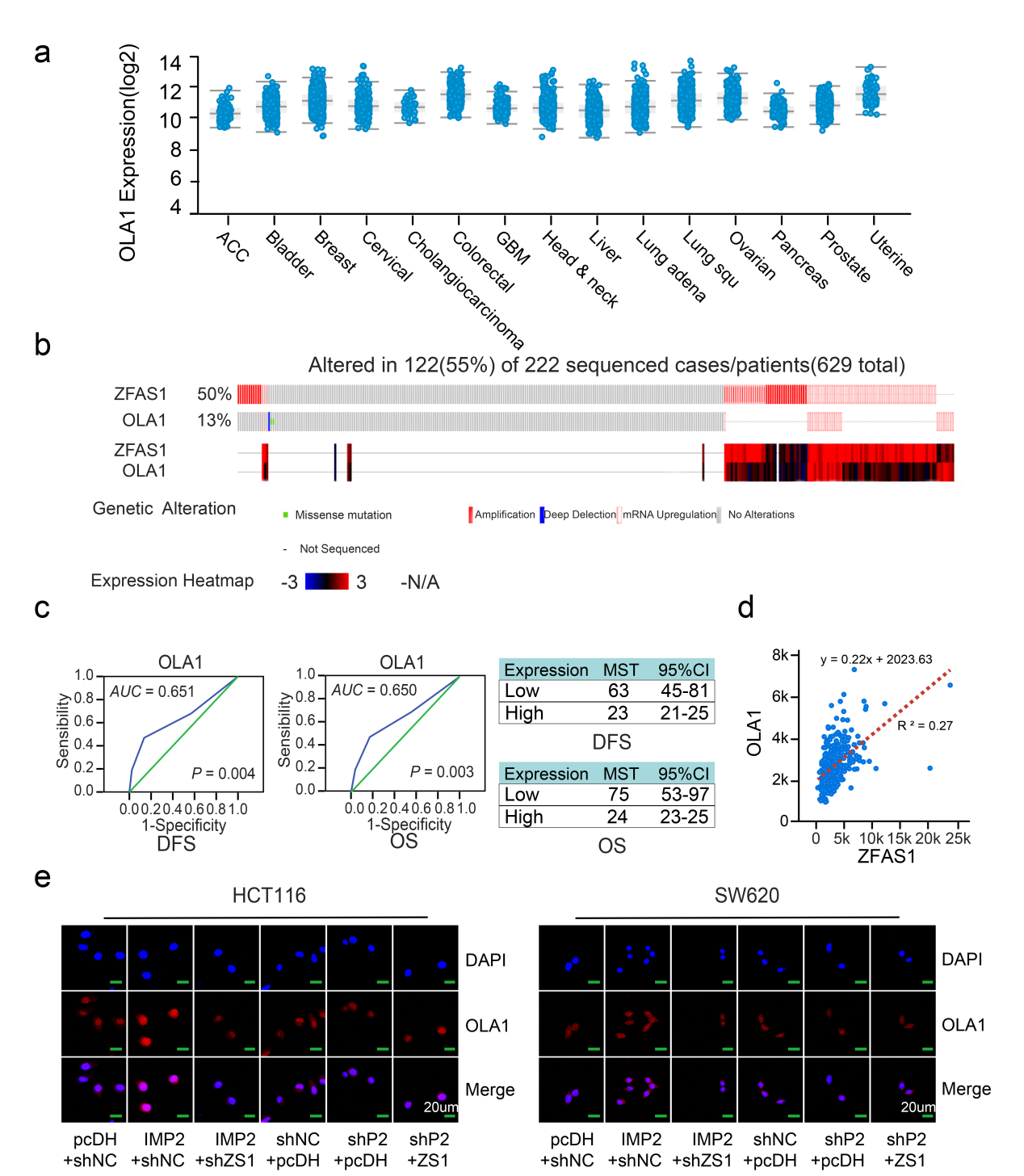
**

**Fig. S4 The relationship of *ZFAS1* with OLA1 expression and clinicopathological features**

**a** Expression of *OLA1* in 15 common cancers in RNA sequencing (RNA-seq) data set platform (http://www.cbioportal.org/). **b** TCGA data showed the gene expression correlation between *ZFAS1* and *OLA1*. **c** The ROC curve method illustrating the cutoff values of OLA1 high/low expression in paired CRC patient tissues vs. adjacent-tumor controls (n=144). **d** Regression analysis of the correlation between *ZFAS1* and *OLA1* in the TCGA dataset. **e** IF method illustrating the protein levels of OLA1 after interfering with *IMP2* alone or interfering with *IMP2* and *ZFAS1* at the same time in HCT116 and SW620 cells.


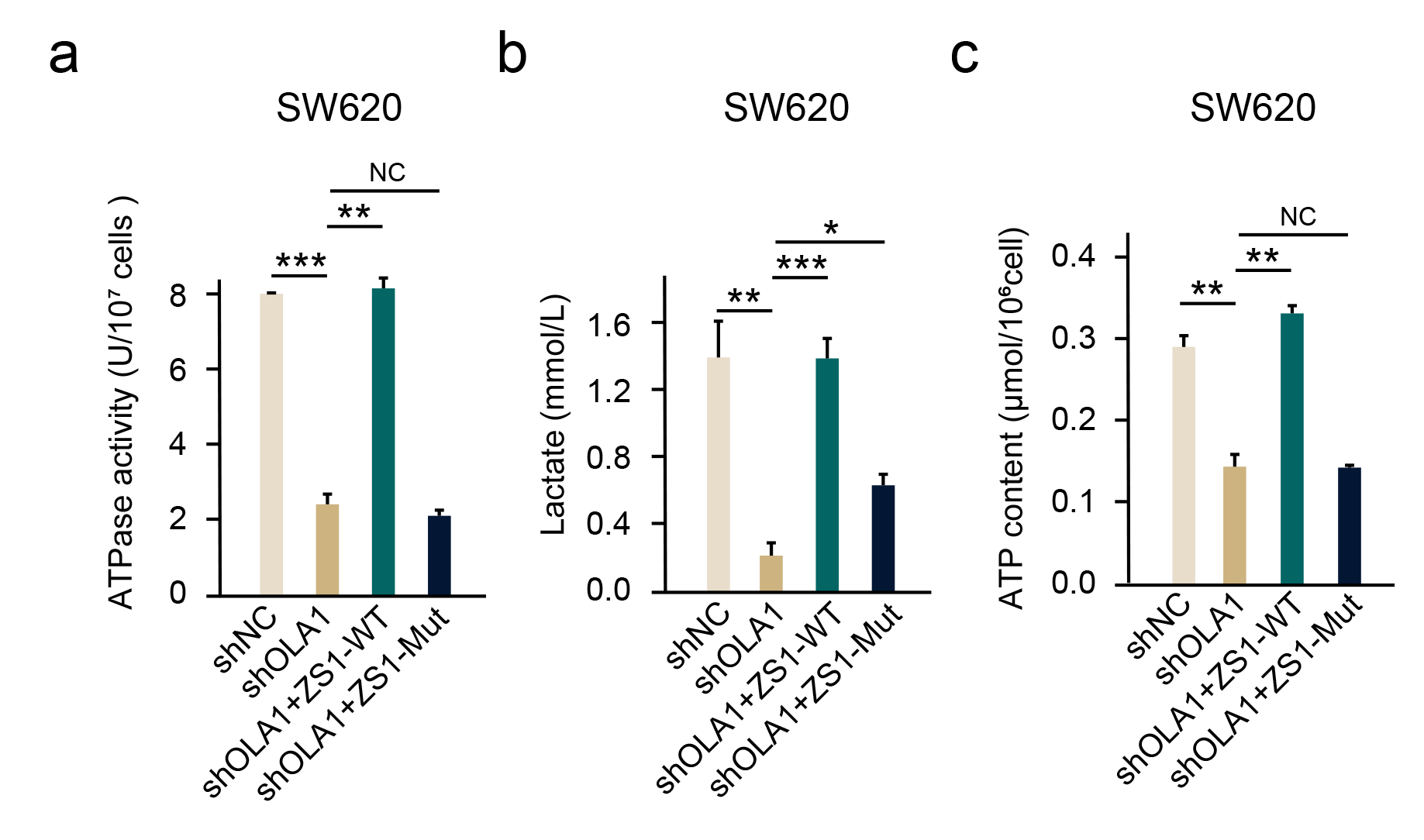


**Fig. S5 Identification of the impact of *ZFAS1*-OLA1 axis on energy metabolism**

**a** Rescue experiments determining the ATPase activity of OLA1 after co-transfected with *shOLA1* and *ZFAS1-Wild/ZFAS1-Mutant* vectors in SW620 cells. **b**, **c** The content of lactate or ATP in the cell supernatant after co-transfected with *shOLA1* and *ZFAS1-Wild/ZFAS1-Mutant* vectors in SW620 cells.


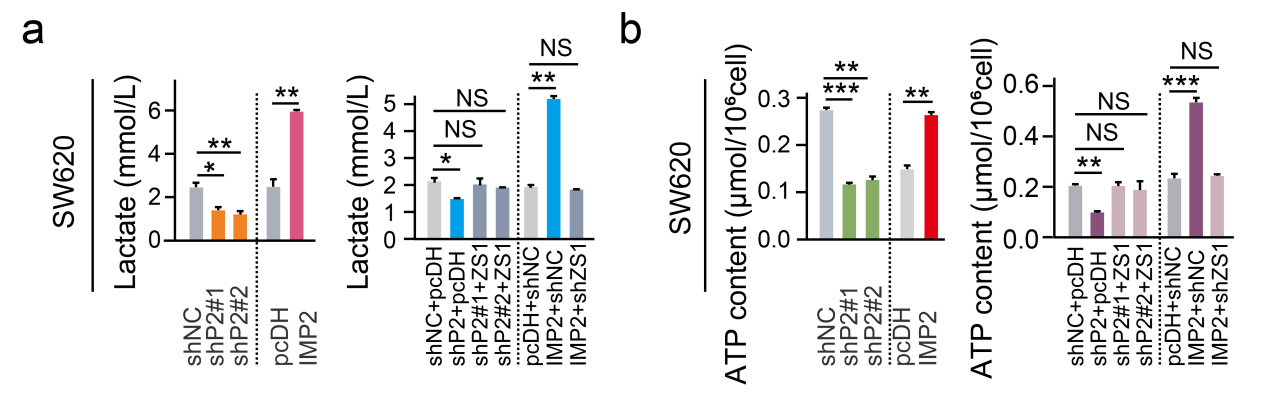


**Fig. S6 Identification of the impact of IMP2-*ZFAS1*-OLA1 axis on energy metabolism**

**a, b** Rescue experiments detecting the lactate content and ATP content after interfering with *IMP2* alone or interfering with *IMP2* and *ZFAS1* at the same time in SW620 cells.
